# A Randomized, Double-Blind, Placebo-Controlled, Parallel-Group, 8-Week Pilot Study of Tuna-Byproduct-Derived Novel Supplements for Managing Cellular Senescence and Cognitive Decline in Perimenopausal and Postmenopausal Women

**DOI:** 10.3390/antiox14050520

**Published:** 2025-04-27

**Authors:** Jintanaporn Wattanathorn, Wipawee Thukham-mee, Terdthai Tong-un, Weerapon Sangartit, Woraluck Somboonporn, Pongsatorn Paholpak

**Affiliations:** 1 Department of Physiology, Faculty of Medicine, Khon Kaen University, Khon Kaen 40002, Thailand; meewep@gmail.com (W.T.-m.); terdthai@kku.ac.th (T.T.-u.); weerasan@kku.ac.th (W.S.); 2 Research Institute for High Human Performance and Health Promotion, Khon Kaen University, Khon Kaen 40002, Thailand; 3 Department of Obstetrics and Gynecology, Faculty of Medicine, Khon Kaen University, Khon Kaen 40002, Thailand; wsomboonporn@yahoo.com; 4 Department Psychiatry, Faculty of Medicine, Khon Kaen University, Khon Kaen 40002, Thailand; ppaholpak@kku.ac.th

**Keywords:** cognitive function, cellular senescence, oxidative stress, inflammation, telomere, SIRT1, menopause

## Abstract

Due to the lack of clinical data, we aimed to assess the anti-cellular senescence and cognition-enhancing effects and the mechanisms of novel tuna-byproduct-derived supplements. An 8-week, three-arm, randomized, double-blind, placebo-controlled parallel study was performed. A total of 60 female perimenopausal and postmenopausal women (45–60 years old) were randomly assigned to placebo, low (2600 mg/day), and high (6000 mg/day) doses of the supplement. The N100 and P300 brain waves, working memory, serum levels of MDA, SOD, CAT, GPx TNF-α, IL-6, eNOS, AChE, MAO, GABA-T, and SIRT1, and density of *Lactobacillus* and *Bifidobacterium* spp. in feces were assessed before consumption and every 4 weeks throughout the study period. The telomere length and total phenolic compound and DHA levels were assessed before and at the end of the study. The low dose increased the N100 amplitude, working memory, telomere length, and SIRT1, whereas high doses improved the amplitudes of N00 and P300, P300 latency, and working memory; suppressed AChE, MAO, and GABA-T; and improved MDA, SOD, GPx, TNF-α, and IL-6 levels in the serum, further exhibiting an increase in DHA. Therefore, the novel supplement could be a potential remedy for managing cellular senescence and cognitive decline in perimenopausal and postmenopausal women; however, studies with larger sample sizes are required.

## 1. Introduction

Cellular senescence is a cellular stress response aimed at the induction of permanent cell cycle arrest. This process can be induced by both extrinsic factors such as oxidative stress and intrinsic factors such as mitochondria dysfunction [1] and telomere shortening [2]. As age advances, senescent cells accumulate, and this change can induce the deterioration of various physiological functions, including age-related reproductive function decline and many associated changes, such as cognitive decline [3]. At ages of over 40 years, almost 50% of women exhibit reproductive aging [2], increasing the risk of memory problems [3,4]. It has been reported that telomere shortening either by oxidative stress or by end-processing events in dividing or non-dividing cells can induce DNA damage, a loss of cell proliferation, senescence, and cell death. In addition, mitochondrial dysfunction—particularly electron transport disturbance—and sirtuin-1 (SIRT1)—an NAD+-dependent histone deacetylase protein—can also accelerate cellular senescence [1,5]. They also play important roles in regulating reproductive aging, affecting the number and quality of oocytes [2,6,7,8] and menopause-related cognitive impairment [9,10,11].

Currently, popular treatment methods for menopause-related symptoms, including cognitive impairment, include hormone replacement therapy (HRT). However, therapeutic efficacy with respect to cognitive impairment is still controversial. Several studies report the benefits of HRT, such as a reduction in cognitive impairment and a decreased risk of Alzheimer’s disease, whereas some studies report no benefits [12]. Furthermore, some adverse effects, such as an increase in breast cancer risk and both venous and pulmonary thromboembolism [13], are reported. Since cognitive function plays a crucial role in the maintenance of quality of life (QoL) [14], the protective effect against cognitive impairment must be considered. Due to the limitations of HRT and the far-reaching impact of cognitive function on QoL together with the concept of “food as medicine”, the beneficial effects of food applications in preventive medicine [15] have gained substantial attention.

Evidence accumulated during this decade has revealed that omega-3 polyunsaturated fatty acids (PUFAs) improve cognition, neuronal preservation, and protection against neurodegeneration [16,17]. Data obtained from clinical trials also reveal that long-chain polyunsaturated omega-3 fatty acids (LC-n3-FA) improve memory performance in terms of the recall ability of object locations in healthy older adults aged between 50 and 75 years old [18]. Furthermore, the consumption of docosahexaenoic acid (DHA) at a dose of 1.16 g DHA per day for 6 months improves the working memory of healthy adults aged between 18 and 45 years old [19]. Our previous study on an animal model of menopause has demonstrated that omega-3-rich tuna oil improves oxidative stress and inflammation and enhances cholinergic functions, giving rise to improvements in memory performances with respect to bilateral ovariectomized rat models [20]. In addition, a recent clinical study clearly demonstrated that children between 6 and 12 years old who consumed fish oil capsules that contained docosahexaenoic acid (DHA)—at doses of 260 and 520 mg once a day for 12 weeks—exhibited improvements in cognitive ability [21]. Owing to these pieces of information and the role of cellular senescence on menopause-related cognitive impairment mentioned earlier, it has been hypothesized that DHA-enriched functional beverages can also improve cellular senescence and menopause-related cognitive decline. Although our preclinical data demonstrate the cognition-enhancing effect of functional beverages containing tuna oil from canned tuna byproducts [20], no available clinical data confirm this benefit. Because many potential supplements in animal models failed to show effects in clinical trials, clinical data that confirm potential benefits are necessary. Moreover, due to the lack of clinical data regarding the cognition-enhancing effect of omega 3-enriched tuna-oil-containing beverages, we aimed to determine the effect of these beverages on cognitive function and the possible underlying mechanisms in postmenopausal women.

## 2. Materials and Methods

### 2.1. Preparation of the Functional Beverage Containing Tuna Oil

The functional beverage containing tuna oil used in this study was kindly provided by Thai Union Group Public Company Limited. The main components comprised soy protein, tuna oil, sunflower seed oil, ground black sesame, and a vitamin B complex, as shown in Table 1. Furthermore, the nutritional facts of the developed product are provided in Table 2. The chromatogram of the fat composition of the tuna-oil-containing functional beverage used in this study is provided in Supplementary Materials File S1. The beverage’s formulation was officially submitted as a Thai petty patent application, TH2203003264.

### 2.2. Study Design

This study was a 3-arm, randomized, double-blind, placebo-controlled study, and it was approved by the Center for Ethics in Human Research, Khon Kaen University (HE641562). The approval date was 26 December 2021. Moreover, it was registered in the Thai Clinical Trial Registry (TCTR20211201001) and was performed in accordance with the International Conference of Harmonization (ICH) for Good Clinical Practice (GCP); it is also in compliance with the Declaration of Helsinki and its further amendments. The study site was set up at the Faculty of Medicine, Khon Kaen University. All subjects provided a consent form before participating.

In this study, perimenopausal and postmenopausal women who met the following criteria were recruited: aged between 45 and 60 years old (<5 years of menstruation cessation); no serious physical and psychological illness; 18 ≤ body mass index (BMI) ≤ 23; possessed good Thai language communication skills; resided in Khon Kaen province, Thailand. The exclusion criteria included the following: (1) chronic physical illness with diabetes, cardiovascular disorders, liver disorder (aspartate transaminase or AST > 64 U/L; alanine transaminase or ALT > 50 U/L), kidney disease, gastrointestinal disorder, hematological disorder, allergy, and cancer; (2) head trauma; (3) hysterectomy or oophorectomy; (4) application of hormone replacement or drugs that affect the function of the hypothalamic–pituitary–adrenal axis (HPA axis) within 3 months prior to participation in the project; (5) application of drugs that affect the nervous system; (6) alcohol addiction (>5 glasses/day) or drug abuse history; (7) smoker (≥10 cigarettes/day); and (8) participation in other projects. After a physical examination carried out by a physician, the enrolled volunteers who fitted the inclusion criteria were randomized via a randomized block design into three groups: placebo, 2600 mg tuna oil beverage per day, and 6000 mg tuna oil beverage per day (n = 20/arm).

In this study, all beverages provided to the volunteers were identical in terms of general characteristics, including color, shape, weight, and flavor, and the packaging was designed to make distinguishing between the tuna oil beverage and the placebo impossible.

**Figure 1:**
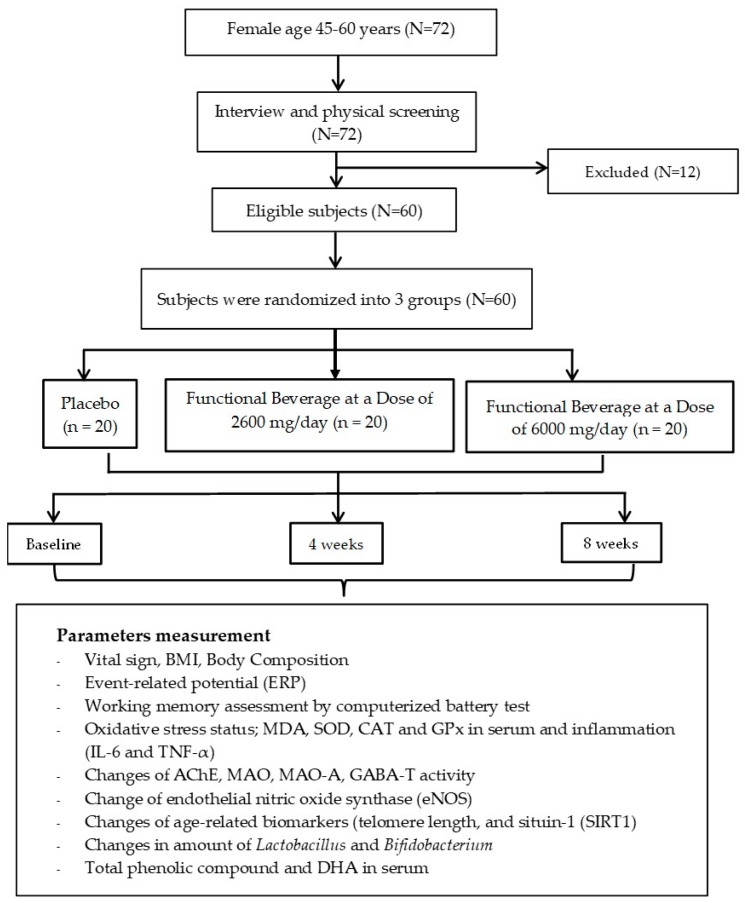
Schematic diagram showing the study’s protocol.

All subjects were supposed to consume the assigned substances for a duration of 8 weeks. Vital signs, body mass index (BMI), cognitive function, working memory, and changes in various parameters—including neurotransmitters (acetylcholine, monoamine, and GABA); inflammatory mediators (IL-6 and TNF-α); oxidative stress markers (malondialdehyde or MDA, superoxide dismutase or SOD, catalase or CAT, and glutathione peroxidase or GPx); age-related markers, such as telomere length; sirtuin-1 (SIRT1); and the density of *Lactobacillus* and *Bifidobacterium* spp.—were assessed at baseline and every 4 weeks until the end of an 8-week study period. Furthermore, safety parameters, including changes in hematological parameters and clinical chemistry values, were also determined every 4 weeks throughout the study period. At the end of the study, docosahexaenoic acid (DHA) levels were also monitored. All experimental procedures are summarized in the schematic diagram shown in Figure 1.

### 2.3. Measurement of Body Mass Index (BMI) and Body Composition

All subjects were instructed to cease food consumption at least for 8 h prior to the measurement. The BMI and body composition were monitored using a bioimpedance body fat analyzer (BC-621, Tanita Co., Tokyo, Japan). Height and weight were recorded for BMI calculations using the following formula: BMI = weight (kg)/height (m^2^) [22].

### 2.4. Event-Related Potential (ERP) Assessment

We assessed cognitive functions using the non-invasive event-related potential (ERP) as a tool. The record was obtained using a 40-channel EEG system (Neuroscan, Inc., Sterling, Herndon, VA, USA). The procedures were elaborated upon in our previous study [23,24]. Briefly, brain activity was assessed via Ag–AgCl electrodes that were placed on the scalp, following the international 10/20 electrode positioning system. The left earlobe was used as the reference electrode, whereas the right earlobe served as the ground electrode. According to the auditory oddball paradigm, the auditory stimulus sequences at 2 different frequency tones—650 Hz and 1 kHz—were delivered to each subject binaurally at 80 dB via electrostatic headphones (NordicNeuroLab, Milwaukee, WI, USA). These were regarded as non-targeted (probability of 80%) and targeted stimuli (probability of 20%), respectively. Brain wave activities in the late response obtained when performing an auditory oddball paradigm task—including N100 or a negative peak occurring between 65 and 135 milliseconds and P300 or a positive peak occurring between 280 and 350 milliseconds—served as indices for attention and cognitive processing, respectively [23,24].

### 2.5. Working Memory Assessment

A group of recognition tests—comprising simple reaction time, choice reaction time, digit vigilance, numeric working memory, word recognition, picture recognition, and spatial memory tests—was implemented for assessing working memory in the form of a computerized battery test. The presentation order was as follows: word recognition, picture presentation, simple reaction time, digit vigilance task, choice reaction time, spatial working memory, and numeric working memory. All tests were provided using a high-resolution VGA color monitor, and responses were recorded via a two-button (yes/no) response box. This group of recognition tests was used to monitor 4 working memory domains, including the power of attention, continuity of attention, speed of memory, and quality of memory. The test–retest reliability in each working memory domain was in a range between 0.72 and 0.82 (power of attention = 0.75, continuity of attention = 0.78, quality of working memory = 0.82, and speed of memory = 0.72) [24,25].

Word recognition test: Each subject was exposed to a set of 15 words. Both the stimulus duration and interstimulus interval were 1 s. Then, this was followed by a recall test. Subjects had to try to recall as many words as possible. Both the time for response and the percentage of accuracy were recorded.

Picture recognition test: In total, 20 photographic images were presented at a rate of 1 image every 3 s to each subject using a monitor, with a stimulus duration of 1 s. The subject had to memorize them and recall them later as much as possible. The reaction time of the subject’s response and the percentage of accuracy were recorded.

Simple reaction time: Each subject was instructed to press the “yes” response button as quickly as possible every time the word “yes” was presented on the monitor. The percentage of accuracy and reaction time were measured.

Digit vigilance task: Each subject was instructed to press the “yes” button as quickly as possible when a target digit that was randomly selected and constantly displayed on the upper right corner of the monitor screen matched a series of digits that were provided at the center of the screen at a rate of 80 min/min. The task lasted 1 min, and there were 15 stimulus–target matches. Both the percentage of accuracy and response time spent to accomplish the task were recorded.

Choice reaction time: In this test, each subject was exposed to a series of 50 words. The words “yes” or “no” were randomly shown on the monitor. The “yes” button was pressed when the presented word was yes, and the “no” button was pressed when the presented word was no. Then, the reaction time or time spent on the response and the percentage of accuracy were recorded.

Spatial working memory: To test visuospatial memory, each subject was exposed to a picture of a house with nine windows, and four of the windows were illuminated for 3 s. The participant had to memorize the position of the illuminated windows as much as possible. Following this step, a series of house pictures were provided, and the subject was instructed to press the “yes” button when the position of the illuminated window in the presented picture corresponded with the first picture; vice versa, the participant was instructed to press the “no” button when the image did not correspond to the position of the illuminated windows. The percentage of accuracy and the reaction time of responses were measured.

Numeric working memory: A group of 5 numbers from 0 to 9 was presented on the monitor, and the subject was instructed to memorize them. Following this step, a series of 30 numbers from 0 to 9 was provided to the subject, and they were instructed to press the “yes” button when the numbers in the first group were presented; when the presented numbers were not in the first group, the “no” button was pressed. The performances mentioned were performed as rapidly as possible, and the response time and the percentage of accurate responses were recorded.

### 2.6. Blood Collection and Preparation

Blood samples were collected from the antecubital vein, and they were separated for collection in 2 types of tubes. Blood collected in a tube containing ethylenediaminetetraacetic acid (EDTA) was prepared as plasma for the determination of telomere lengths, whereas blood collected in tubes without EDTA was used for serum preparation and various analyses. For plasma preparation, the collected blood, which was not allowed to clot, was centrifuged at 2000× *g* for 10 min in order to remove cells. The supernatant was harvested and served as plasma. For serum preparation, blood in the tubes was allowed to clot at room temperature for 30 min. Then, these samples were centrifuged at 1000–2000× *g* for 10 min in a refrigerated centrifuge. Following this process, the liquid component or serum was transferred to a clean microcentrifuge tube and kept at −20 °C until it was used.

### 2.7. Neurotransmitter Assessment

#### 2.7.1. Acetylcholinesterase (AChE) Activity

AChE measurements were performed using the colorimetric method. This method was modified from a previous method described elsewhere [24,25,26]. Briefly, the reaction mixture consisting of 25 µL of 15 mM ATCI, 75 µL of 3 mM DTNB, and 50 µL of 50 mM Tris-HCl—pH 8.0, containing 0.1% bovine serum albumin (BSA)—was mixed with 25 µL of the serum using a 96-well microplate. Then, the mixture was subjected to a 5 min incubation period. At the end of the incubation period, absorbance at 415 nm was measured (iMark™ Microplate Absorbance Reader, Bio-Rad Laboratories, Inc., Hercules, CA, USA). Following this step, 10 µL of acetylcholine thiochloride (ACTI) was added and incubated for 3 min. At the end of incubation, absorbance at 415 nm was measured, and AChE activity was calculated according to the equation below and expressed as nmol/mg protein:

AChE activity = (ΔA/1.36 × 10^4^) × 1/(120/230)C

ΔA: difference in absorbance/minute; C: protein concentration of brain homogenate protein.

#### 2.7.2. Monoamine Oxidase (MAO) Levels

MAO assays were performed via the colorimetric method. Briefly, a chromogenic mixture consisting of 1 mM vanillic acid (Sigma-Aldrich, St. Louis, MO, USA), 500 µM 4-aminoantipyrine (Sigma-Aldrich, MO, USA), and 4 U/mL peroxidase (Sigma-Aldrich, MO, USA)—which was dissolved in a 0.2 M potassium phosphate buffer with a pH of 7.6—was mixed with an aliquot of the serum at a volume of 50 µL using a 96-well microplate. After mixing thoroughly, 50 µL of the chromium solution and 200 µL of tyramine 500 µM P-Tyramine (Sigma-Aldrich, MO, USA) were added, and the sample was incubated at 37 °C for 30 min. At the end of the incubation period, an optical density of 490 nm was recorded using a microplate reader (iMark™ Microplate Absorbance Reader, Bio-Rad Laboratories, Inc., Hercules, CA, USA). All data values were expressed as µmol/mg protein [27].

#### 2.7.3. Gamma-Aminobutyric Acid-Transaminase (GABA-T) Activity

GABA-T measurements were carried out via the colorimetric method. Briefly, the reaction mixture consisting of 20 mM gamma-aminobutyric acid (GABA) (Sigma-Aldrich, MO, USA), 10 mM α-ketoglutarate (Sigma-Aldrich, MO, USA), and 0.5 mM NAD (Sigma-Aldrich, MO, USA) in a sodium phosphate buffer (0.05 mM, pH 8.0) was mixed with an aliquot of plasma at volumes of 100 µL and 200 µL. After mixing, the mixture was incubated at 37 °C for 30 min. At the end of incubation, the absorbance at 340 nm was read using a microplate reader (iMark™ Microplate Absorbance Reader, Bio-Rad Laboratories, Inc., Hercules, CA, USA) [28,29].

### 2.8. Endothelial Nitric Oxide Synthase (eNOS) Assessment

eNOS activity assessment was performed using a human ELISA kit (MBS265088, MyBioSource, San Diego, CA, USA). Briefly, the standard solution, blank, and tested sample at concentrations of 50 µL, 50 µL, and 40 µL were added to the microplate. Following this step, 10 µL of the biotinylated antibody was mixed with the tested sample and blank. Then, an aliquot of streptavidin-HRP at a volume of 50 µL was mixed with the standard and the tested sample. The plate was covered and incubated at 37 °C for 1 h. At the end of incubation, the microplate was washed 5 times with 300 µL of a washing buffer. After washing, 50 µL of substance solution A was added, followed by 50 µL of substance solution B. Then, they were mixed thoroughly, and they were incubated at 37 °C for 10 min. At the end of incubation, the reaction was terminated by adding 50 µL of the stop solution. Then, the absorbance at 450 nm was measured within 10 min using a microplate reader (iMark™ Microplate Absorbance Reader, Bio-Rad Laboratories, Inc., Hercules, CA, USA).

### 2.9. Oxidative Stress Status Assessment

The oxidative stress status was monitored using malondialdehyde (MDA), a product of the lipid peroxidation process. The activities of the main scavenger enzymes, such as superoxide dismutase (SOD), catalase (CAT), and glutathione peroxidase (GSH-Px), were used as indices reflecting the oxidative stress status.

#### 2.9.1. Malondialdehyde (MDA) Measurement

Thiobarbituric acid reactions were used to assess malondialdehyde (MDA), which is a product of the lipid peroxidation process [30]. The mixture solution containing 50 µL of 8.1% sodium dodecyl sulfate (Sigma-Aldrich, MO, USA), 375 µL of 0.8% thiobarbituric acid (Sigma-Aldrich, MO, USA), 375 µL of 20% acetic acid (Sigma-Aldrich, MO, USA), and 150 µL of distilled water was mixed with an aliquot of the serum at a volume of 50 µL. The mixture was mixed thoroughly and heated at 95°C for 60 min. Then, the mixture was cooled with tap water and mixed with 1250 µL of a mixture of n-butanol and pyridine at a ratio of 15:1 (Merck, Darmstadt, Germany). After mixing thoroughly, the mixture was centrifuged at 4000 rpm for 10 min. At the end of the centrifugation period, the upper layer was harvested, and the absorbance at 532 nm was determined. The standard was prepared using 1,1,3,3-tetramethoxy propane (0–15 µM) (Sigma-Aldrich, MO, USA). The level of MDA was expressed as µg/mg protein.

#### 2.9.2. Superoxide Dismutase (SOD) Activity Assessment

SOD activity was assessed using the nitroblue teterazolium (NBT) method. Briefly, an aliquot of the serum at a volume of 20 µL was mixed with 200 µL of the reaction mixture consisting of a 57 mM phosphate-buffered solution (KH_2_PO_4_) (Sigma-Aldrich, MO, USA), 0.1 mM EDTA (Sigma-Aldrich, MO, USA), 10 mM cytochrome c (Sigma-Aldrich, MO, USA), and 50 µM of xanthine (Sigma-Aldrich, MO, USA). Then, 20 µL of the xanthine oxidase solution at a concentration of 0.90 mU/mL (Sigma-Aldrich, MO, USA) was added. Following this step, the absorbance at 415 nm was measured. SOD enzyme (Sigma-Aldrich, MO, USA) activities at concentrations ranging from 0 to 25 units/mL were used as the standard, and SOD activities were expressed as units/mg protein [20,31].

#### 2.9.3. Catalase (CAT) Activity Assessment

Based on the crucial role of catalase in defending against both exogenous and endogenous H_2_O_2_, particularly at low concentrations, we also measured CAT activity in this study [32] via the decomposition of H_2_O_2_. A serum aliquot at a volume of 10 µL was mixed with a reaction solution comprising 50 µL of 30 mM hydrogen peroxide in a 50 mM phosphate buffer at a pH of 7.0 (BDH Chemicals Ltd., London, UK), 25 µL of H_2_SO_4_ (Sigma-Aldrich, MO, USA), and 150 µL of 5 mM KMnO_4_ (Sigma-Aldrich, MO, USA). Then, absorbance at 490 nm was measured. The standard curve was prepared using CAT enzymes (Sigma-Aldrich, MO, USA) at concentrations ranging from 0 to 100 units/mL. CAT activity was expressed as units/mg protein [22,32,33].

#### 2.9.4. Glutathione Peroxidase (GPx) Activity

GPx activity plays a crucial role in changing H_2_O_2_ to H_2_O, particularly at high concentrations of H_2_O_2_ [31]; thus, we also measured this type of enzyme activity. Briefly, 20 µL of the sample was mixed with a reaction mixture consisting of 10 µL of 1 mM dithiothreitol (DTT) (Sigma-Aldrich, MO, USA) in a 6.67 mM potassium phosphate buffer (Sigma-Aldrich, MO, USA) (pH 7), 100 µL of 1 mM sodium azide (Sigma-Aldrich, MO, USA) in a 6.67 mM potassium phosphate buffer (Sigma-Aldrich, MO, USA) (pH 7), 10 µL of a 50 mM glutathione solution (Sigma-Aldrich, MO, USA), and 100 µL of 30% hydrogen peroxide (BDH Chemicals Ltd., London, UK). The mixture was mixed thoroughly and incubated at 25 °C for 5 min. At the end of the incubation period, an aliquot of 10 mM DTNB (5,5′-dithiobis(2-nitrobenzoic acid) (Sigma-Aldrich, MO, USA)) at a volume of 10 µL was added, and the absorbance at 412 nm was measured at 25 °C for 5 min. GPx enzymes (Sigma-Aldrich, MO, USA) at concentrations ranging from 0 to 5 units/mL were used for preparing a standard calibration curve. GPx activity was expressed as units/mg protein [23,34].

### 2.10. Inflammatory Cytokine Assessment

The tumor necrosis factor-alpha (TNF-α) ELISA kit (ab108908 from Abcam Limited., Cambridge, UK) was assessed according to the guideline provided by the manufacturing company and a method previously used for assays by our team [20,34]. Briefly, 50 µL of the serum solution (1:10) was incubated in a shaking microplate for 2 h. At the end of the incubation period, the samples were washed 5 times with a 1× wash buffer at a volume of 200 µL/well. Then, 50 µL of a biotinylated TNF-α detector antibody was added. The mixture was incubated in the shaking microplate for 2 h and again washed 5 times with a 1× wash buffer at a volume of 200 µL/well. After washing, 50 µL of conjugated streptavidin–peroxidase was added to the mixture in each well, and the mixture was incubated for 30 min. At the end of incubation, the mixture in each well was washed again 5 times with 200 µL of the wash buffer. Following this step, the mixture was mixed with 50 µL of a chromogen substrate and subjected to a 20 min incubation period. The reaction was stopped with a stop solution at a volume of 50 µL/well. The absorbance at 40 nm was recorded using an ELISA reader (Sunrise™, Tecan Trading AG, Männedorf, Switzerland).

The detection of interleukin-6 (IL-6) levels was performed using a human IL-6 ELISA kit (ab178013 from Abcam Limited, Cambridge, UK) according to guidance provided by the manufacturing company and the method previously used for the assay by our team [35]. Briefly, 100 µL of the sample solution was incubated in a microplate well at 37 °C for 90 min, mixed with 100 µL of a biotinylated antibody, and subjected to incubation at 37 °C again but for 1 h. The sample in each well was washed with a wash buffer solution at a volume of 300 µL and mixed with a TMB substrate at a volume of 100 µL/well. Then, all samples were incubated at 37 °C for 30 min. Then, the reaction was stopped using 100 µL of the stopping solution per well. The absorbance at 450 nm was recorded within 10 min with an ELISA reader (Sunrise™, Tecan Trading AG, Männedorf, Switzerland) [22].

### 2.11. Age-Related Biomarker Assessment

#### 2.11.1. DNA Extraction and Assessment of Telomere Length

Based on the information regarding the validity of using DNA extracted from human plasma in polymerase chain reactions (PCRs) [36], the DNA of all subjects was extracted from their plasma using a GF-1 nucleic acid extraction kit (Vivantis Technologies Sdn Bhd, Selangor Darul Ehsan, Malaysia). To induce blood lysis, 200 µL of Buffer BB was added into a 200 µL plasma sample in a microcentrifuge tube. After mixing thoroughly with a vortex, 20 µL of Proteinase K was added, mixed immediately, and incubated at 65 °C for 10 min. Following this step, 20 µL of RNase A (DNase-Free, 20 mg/mL) was added, mixed, and incubated at 37 °C for 10 min to remove RNA. Then, 200 µL of absolute ethanol was added and mixed thoroughly to produce a homogeneous solution. The solution was loaded into the column, assembled in a provided clean collection tube, and subjected to a 5000× *g* centrifugation process for 1 min. The flow trough was discarded. After this process, the first washing process was started by washing the column with 500 µL of wash buffer 1, which was subjected to 5000× *g* centrifugation for 1 min. Again, the flow trough was discarded. The column was subjected to the second wash with 500 µL of wash buffer 2 and centrifuged at 5000× *g* for 1 min. The flow through was discarded. Following this step, a column was rewashed with 500 µL of wash buffer 2, and it was centrifuged at maximum speed for 3 min. To elute DNA from the column, the column was placed in a clean microcentrifuge tube; 100 µL of the preheated elution buffer, TE buffer, or sterile water was eluted directly onto the column membrane and allowed to stand for 2 min; centrifugation was carried out at 5000× *g* for 1 min to elute DNA. Then, DNA was quantified using a Thermo Scientific^TM^ NanoDrop spectrophotometer (Fisher Scientific, Portsmouth, NH, USA). The DNA extraction can be stored at −80 °C until used.

The PCR mixture was also freshly prepared. Briefly, the mixture for the amplification reaction comprised 2.5 µL of 25 mM MgCl_2_; 2.5 µL of 2.5 mM dNTP; 2.5 µL of the PCR buffer; 0.5 µL of Taq-polymerase (5 U/µL); 0.7 µL (10 mM/L) of each telomere primer such as telomere forward (*teloF*) (Integrate DNA, Technolgies, Coralville, IA, USA) and telomere reward (*teloR*) (Integrate DNA, Technolgies, Coralville, IA, USA); and single-copy genes such as 36B4 Forward (*36B4F*)(Integrate DNA, Technolgies, Coralville, IA, USA) and 36B4 Reward (*36B4R*) (Integrate DNA, Technolgies, Coralville, IA, USA). In this study, 36B4 served as a reference gene encoding acidic ribosomal phosphoproteins. The detailed nucleotides of all mentioned primers are shown in Table 3. Then, the PCR mixture was mixed with 0.3 µL of the SYBR^®^ Green PCR Master Mix (2×) (Thermofisher Scientific, MA, USA), 14 µL of PCR-grade water, and 2 µL of DNA (5 ng/uL sample). A real-time system (Roter-Gene, QIAGEN, Germantown, MD, USA) was implemented for the amplification process. The thermal cycling of *36B4* included 1 cycle (95 °C for 15 s) followed by 40 cycles (95 °C for 15 s and 57 °C for 1 min), whereas telomere cycling included 1 cycle (95 °C for 10 min) followed by 50 cycles (95 °C for 15 s and 58 °C for 1 min) [36,37,38].

The telomere length (TL) was calculated using the provided equation:

TL (kb) = 3.274 + 2.413 × (T/S)

The T/S ratio or telomere gene/single-copy gene ratio can be calculated from the cycle threshold (Ct) of the telomere and single-copy gene (36B4) according to the following formula previously described [37,38]:

ΔCt telomere = [Ct (telomere of DNA sample) − Ct (telomere of DNA control)]

∆Ct single copy gene= [Ct (single copy gene of DNA sample) − Ct (single copy gene of DNA control)]

∆∆Ct sample = [∆Ct telomere − ∆Ct single copy gene]

Relative telomere (T/S ratio) = 2^−∆∆Ct^ sample

Detailed of genes used in this study was provided in Supplementary File S5.

#### 2.11.2. Assessment of Situin-1 (SIRT1)

Owing to the crucial role of sSIRT1—an NAD+-dependent deacetylase—in regulating age-related cellular processes and neurodegeneration [39], we also determined the effect of tuna oil beverages on the alteration of SIRT1. The determination of SIRT1 was performed using a human Sirtuin 1 Elisa Kit (my bioresource Cat No. MBS161084, San Diego, CA, USA). Briefly, 10 µL of the serum was incubated in each well of a microplate at 37 °C for 1 h. At the end of incubation, it was washed with 300 µL of a buffer solution 2 times and incubated with 100 µL of the biotinylated antibody at 37 °C for 1 h. Then, it was washed 3 times with 300 µL of a wash buffer solution. After washing, 100 µL of the conjugated enzyme was added to each well and incubated at 37 °C for 30 min. Following this step, an aliquot of a color reagent at a volume of 100 µL was added to stop reactions, and the absorbance at 450 nm was measured within 10 min using an ELISA reader (Sunrise™, Tecan Trading AG, Männedorf, Switzerland).

### 2.12. Lactobacillus and Bifidobacterium spp. Assessment

The determination of *Lactobacillus* and *Bifidobacterium* spp. was performed according to previously described steps [40]. Briefly, 0.1 mL of the sample was prepared as 10-fold serial dilutions. Then, 0.1 mL of each dilution was added to a De Man, Rogosa, and Sharpe (MRS) agar and Bifidobacterium agar (Himedia™, Mumbai, India) to carry out the standard spread plate method (in duplicated plates). Then, the plates were incubated in anaerobic conditions in a jar with a volume of 2.5 L containing a sachet, generating a carbon dioxide atmosphere (BD GasPak™ EZ, Silver Spring, MD, USA) at 37 °C for 48 h. The amount of *Lactobacillus* spp. and *Bifidobacterium* spp. colonies on the MRS agar and Bifidobacterium plates was reported as the log value of the colony-forming unit (log CFU/mL).

### 2.13. Determination of Docosahexaenoic Acid (DHA)

To extract lipids from the sample, an aliquot of the sample at a volume of 300 µL was mixed with a mixture of hexane and isopropanol (3:2, *v*/*v*) at a volume of 1000 µL. After mixing, it was stored at −20 °C for 15 min. Then, it was centrifuged at 4 °C at 10,000 rpm for 10 min. The supernatant was collected and dried via nitrogen. The dried sample was dissolved in 1 mL of 80% methanol and filtered with a syringe filter at a diameter of 0.22 µm prior to analyses.

The level of DHA was determined using a liquid chromatography–mass spectrometry (LC–MS/MS) system comprising an LCMS-8030 triple quadrupole mass spectrometer (Shimadzu, Kyoto, Japan) operated in the electrospray ionization (ESI) mode and a Shimadzu LC-20AC series HPLC system (Shimadzu, Kyoto, Japan). The LC system was operated using the isocratic mode, and separation was carried out with a C18 column with acetonitrile (solvent A) and 2 mM of ammonium acetate (solvent B) (90%:10%) as mobile phases at a flow rate of 0.3 mL/min. The sample injection volume was 2 µL.

A triple quadrupole mass spectrometer (Shimadzu, Kyoto, Japan), operated using an electrospray ionization (ESI) source in the negative mode, was used in this study. The system was set up as follows: an interface temperature of 350 °C, a desolvation line of 250 °C, and a heat block temperature of 400 °C. The system was maintained at an interface voltage of 3.5 kV. Nitrogen was used as a nebulizing gas at a flow of 3 L/min. The nebulizing gas flow was maintained at 3 L/min, whereas the drying gas flow was maintained at a rate of 15 L/min. The measurement was carried out using the modified method of Serafim and colleagues [41].

### 2.14. Determination of Total Phenolic Compounds (TPC) in Serum

The level of TPC in serum was assessed using a modified Folin–Ciocalteu (F-C) regeant [42]. The serum was extracted with perchloric acid (70%, 1:1 *v*/*v*) and centrifuged at 2700× *g* at 4 °C for 10 min to remove the precipitated proteins. Then, an aliquot of serum at a volume of 50 mL was mixed with 250 mL of the Folin–Ciocalteu reagent (1 N) and agitated via sonication for 5 min. Following this step, 250 mL of 20%Na_2_CO_3_ was added and mixed thoroughly. Then, deionized water was added to the mixture, totaling 2 mL, and the mixture was incubated in the dark at room temperature for 2 h. At the end of incubation, absorbance was measured at 760 nm, and the results were reported in mg of gallic acid equivalents (GAEs) per liter of serum.

### 2.15. Statistical Analysis

Data were presented as mean ± S.D. Statistical analyses were performed using SPSS statistics version 21.0 via testing for normal distributions relative to the Shapiro–Wilk normality test. Data that exhibited normal distributions were assessed using repeated measurement ANOVA followed by LSD post hoc tests; an exception of this method was that DHA data were assessed using ANOVA, followed by LSD post hoc tests. Data that were not normally distributed and DHA levels were analyzed using the Kruskal–Wallis test. Significance was considered when the *p*-value was <0.05.

## 3. Results

Figure 1 shows a summarized flow diagram of the subject throughout the study. A total of 72 perimenopausal and postmenopausal women were recruited to participate in this study. After physical screening was carried out by the doctor, 60 subjects fitted the inclusion criteria, and they were willing to provide consent forms before participating in this project. The eligible subjects were randomized and assigned to the placebo, 2600 mg/day tuna oil beverage, and 6000 mg/day tuna oil beverage groups. No subjects withdrew throughout the 8-week study period.

### 3.1. Demographic Characteristics of the Subjects

Table 4 shows the demographic characteristics of the subjects in this study. It was observed that there were no significant differences in age, vital signs (temperature, blood pressure, and respiratory rate), body weight, height, and body mass index (BMI) among the three groups mentioned earlier. Therefore, it was confirmed that the randomization was successful. These parameters did not exhibit significant differences throughout the study period.

### 3.2. Changes in Brain Wave Components

Table 5 demonstrates that there were no significant differences with respect to amplitude and the latency of N100 and P300 waves relative to the Fz and Cz locations before starting the intervention. After 4 weeks of consumption, subjects who consumed the functional beverage containing tuna oil at a dose of 6000 mg/day exhibited an increase in P300 amplitudes with respect to Fz and Cz (*p*-value < 0.05 for all; compared to the placebo-treated group). With respect to Cz, subjects who consumed the functional beverage at doses of 2600 and 6000 g/day exhibited a significant increase in the N100 amplitude (*p*-value < 0.01 and 0.05, respectively, compared to the placebo-treated group). When the consumption of the assigned substance was prolonged to 8 weeks, subjects who consumed a high dose of the functional beverage exhibited a significant increase in P300 amplitude but decreased P300 latency (*p*-value < 0.05 for all; compared to the placebo-treated group) relative to Fz. The significant increase in the P300 amplitude was also observed in subjects who consumed a low dose of the functional beverage (*p*-value < 0.05; compared to the placebo-treated group) relative to Fz. No other significant changes in the parameters mentioned were observed relative to both Fz and Cz after 4 and 8 weeks of consumption.

### 3.3. Working Memory Assessment via Recognition Tests

We also explored the effect of the tuna-oil-containing functional beverage on working memory using the computerized battery test consisting of various recognition tests, including simple reaction time, choice reaction time, digit vigilance, word recognition, numeric recognition, picture recognition, and spatial recognition tests. All results are shown in Table 6. After the 4-week consumption period, subjects who consumed low doses of the functional beverage exhibited significantly decreased reaction times in the simple reaction time test (*p*-value < 0.05; compared to the placebo-treated group), whereas subjects who consumed a high dose of the functional beverage exhibited a significant reduction in reaction times in the simple reaction time test and word recognition test (*p*-value < 0.01 and 0.05, respectively, compared to the placebo-treated group) and a significant increase in the percentage of accurate responses relative to the word recognition test (*p*-value < 0.05; compared to the placebo-treated group). When the consumption period was prolonged to 8 weeks, subjects who consumed a high dose of the functional beverage exhibited significantly decreased reaction times in the word recognition test and spatial time test (*p*-value < 0.05 and 0.01, respectively, compared to the placebo-treated group). In addition, a significant reduction in spatial test results was also observed in subjects who consumed a low dose of the functional beverage (*p*-value < 0.05; compared to the placebo-treated group). No other significant changes were observed.

### 3.4. Changes in Neurotransmitters

**Figure 2:**
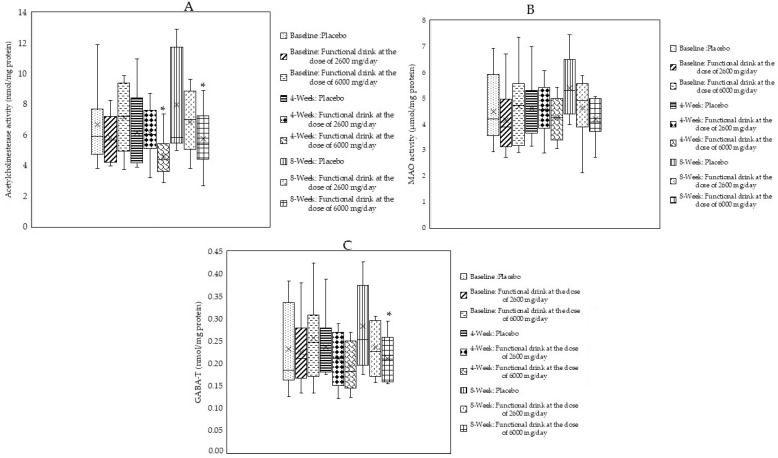
Changes in acetylcholine, monoamine transmitters, and GABA assessed by determining the activities of acetylcholinesterase (AChE) (**A**), monoamine oxidase (MAO) (**B**), and gamma-aminobutyric acid-transaminase (GABA-T) (**C**) in the serum of subjects who consumed the placebo or the functional beverage containing tuna oil at doses of 2600 and 6000 mg per day at baseline and after 4 and 8 weeks of consumption (N = 20/arm). Data are expressed as mean ± SD. * *p*-value < 0.05; compared to the placebo-treated group.

To assess the alterations of acetylcholine, monoamine transmitter, and GABA, indirect assessments via the suppression effect on AChE, MAO, and GABA were applied. After 4 weeks of consumption, subjects who consumed the functional beverage at a dose of 6000 mg per day exhibited significantly decreased AChE (*p*-value < 0.05; compared to the placebo-treated group), as shown in Figure 2A. When the intervention was prolonged to 8 weeks, significant reductions in AChE, MAO, and GABA activities were observed in subjects who consumed the functional beverage at a dose of 6000 mg per day (*p*-value < 0.05 for all; compared to the placebo-treated group), as shown in Figure 2A–C.

### 3.5. Endothelial Nitric Oxide Synthase (eNOS)

**Figure 3:**
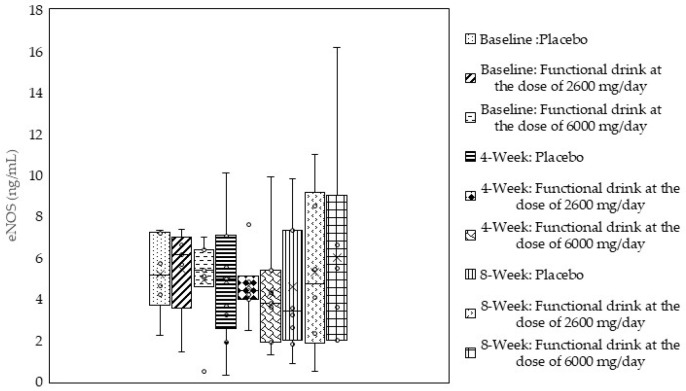
Changes in endothelial nitric oxide synthase (eNOS) levels in the serum of subjects who consumed the placebo or the functional beverage containing tuna oil at doses of 2600 and 6000 mg per day. At baseline and after 4 and 8 weeks of consumption (N = 20/arm). Data are expressed as mean ± SD.

Due to the pivotal effect of the vascular blood supply on cognition [43], we also measured the effect of the functional beverage on eNOS, an important factor that plays a role in the regulation of blood supplies via the role of nitric oxide [44]. The results are demonstrated in Figure 3. It was found that no significant changes in eNOS were observed.

### 3.6. Changes in Oxidative Stress Status and Inflammation

To assess the oxidative stress status, MDA, CAT, and GPx were used as indices reflecting the oxidative stress status, and the data are shown in Table 7. After 4 weeks of consumption, subjects who consumed the tuna-oil-containing functional beverage at doses of 6000 mg per day exhibited significantly decreased MDA but increased GPx activity in their serum (*p*-value < 0.05 for all; compared to the placebo-treated group). When the consumption period was prolonged to 8 weeks, subjects who consumed a high dose of the functional beverage also exhibited a significant increase with respect to SOD activity in their serum (*p*-value < 0.05; compared to the placebo-treated group). However, no other changes were observed in the 8-week study period.

Table 6 also shows changes in inflammatory cytokines such as TNF-α and IL-6. Subjects who consumed a high dose of the functional beverage revealed a significant reduction in TNF-α in the serum after 4 weeks of consumption (*p*-value < 0.05; compared to the placebo-treated group). A significant decrease was noted in both TNF-α and IL-6 in the serum of those who consumed the high-dose functional beverage after an 8-week consumption period (*p*-value < 0.05 for all; compared to the placebo-treated group).

### 3.7. Changes in Age-Related Biomarkers

**Figure 4:**
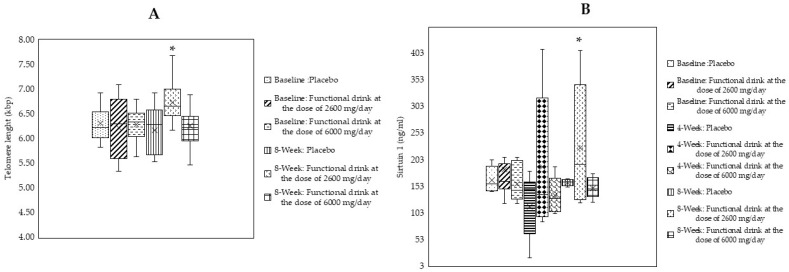
Changes in telomere length (**A**) and in the level of sirtuin-1 or SIRT1 (**B**) of subjects who consumed the placebo or functional beverage at doses of 2600 and 6000 mg per day at baseline and after 4 and 8 weeks of consumption (N = 20/arm). Data are expressed as mean ± SD. * *p*-value < 0.05 compared to the placebo-treated group.

The effects of the functional beverage on alterations in age-related biomarkers—such as telomere length and situin-1 (SIRT1)—were assessed, and the data are shown in Figure 4A,B. It was observed that after 4 weeks of consumption, subjects who consumed high doses of the functional beverage exhibited significant improvements with respect to telomere length and the levels of SIRT1 (*p*-value < 0.05 compared to the placebo-treated group). When the treatment was prolonged to 8 weeks, there were no significant changes in the parameters mentioned in any groups.

### 3.8. Changes in the Amount of Lactobacillus and Bifidobacterium spp.

**Figure 5:**
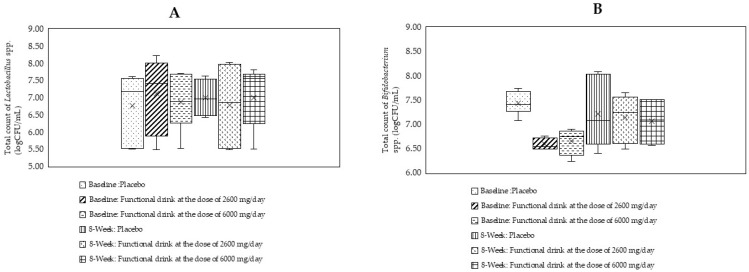
Changes in the density of *Lactobacillus* spp. (**A**) and *Bifidobacterium* spp. (**B**) in the feces of subjects who consumed the placebo or functional beverage at doses of 2600 and 6000 mg per day at baseline and after 4 and 8 weeks of consumption (N = 20/arm). Data are expressed as mean ± S.D.

Figure 5A,B reveal changes in both the above-mentioned species of bacteria. Our data failed to show significant changes in *Lactobacillus* and *Bifidobacterium* spp. throughout the study period.

### 3.9. Levels of Total Polyphenolic Compounds and Docosahexaenoic Acid (DHA) in Serum

**Figure 6:**
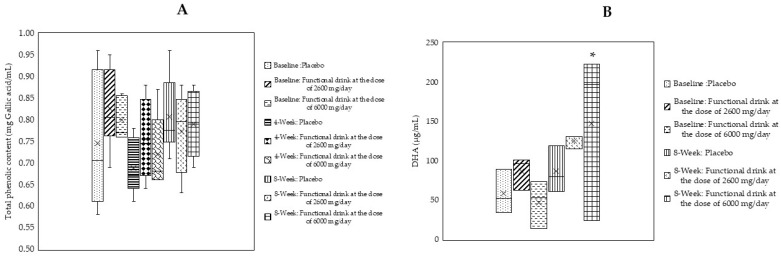
Changes in total phenolic compounds (**A**) and docosahexaenoic acid (DHA) (**B**) in the plasma of subjects who consumed the placebo or functional beverage at doses of 2600 and 6000 mg per day at baseline and after 4 and 8 weeks of consumption (N = 20/arm). Data are expressed as mean ± SD. * *p*-value < 0.05 compared to the placebo-treated group.

The serum levels of the total polyphenolic compounds and docosahexaenoic acid (DHA) were also measured, and the results are shown in Figure 6A,B. Our results demonstrated that no significant changes in the total phenolic compounds levels were observed throughout the study period, whereas the DHA levels in subjects who consumed high doses of the functional beverage exhibited significant increases after 8 weeks of consumption (*p*-value < 0.05 compared to the placebo-treated group).

## 4. Discussion

This study demonstrates that the 8-week consumption of a tuna-oil-containing functional beverage at the doses of 2600 and 6000 mg/day can increase the amplitude of N100, improving performances with respect to simple reaction times and spatial memory tests. However, an increase in the P300 amplitude and improvements in the word recognition test are observed only in subjects who consumed functional beverages containing a high dose of tuna oil. Furthermore, subjects in this group also exhibit a reduction in AChE, MAO, MDA, TNFα, and IL-6 but an increase in GPx activity, whereas subjects who consumed the tuna-oil-containing functional beverage at a dose of 2600 mg/day exhibit improvements with respect to telomere length and SIRT1.

The amplitude of the N100 wave component in the event-related potential (ERP) is associated with selective attention [45], whereas the amplitude of the P300 wave component in ERP is associated with working memory functioning [46]. The increase in the mentioned parameters reflects an increase in the synchronization operations of the neurons contributing an important role in selective attention [47] and working memory capacity [48]. The current data demonstrate that both doses of the tuna beverage promote an increase in N100 amplitude, whereas high doses (6000 mg/day) of the tuna beverage also promote P300 after 4 weeks of consumption. When consumption is prolonged to 8 weeks, both doses also promote an increase in P300 amplitude, and high doses also decrease P 300’s latency. These changes suggest an improvement in the synchronization of neurons that play pivotal roles with respect to selective attention and working memory. It has been reported that P300 latency reflects the speed of cognitive processing in the brain [47,48], which depends on myelination [48]. The improvements detected using brain waves such as N100 and P300 correspond to improvements in simple reaction time, word recognition, and spatial memory tests. In this study, the significant increase in the N100 amplitude disappeared when the participant was re-exposed to the same circumstance during a second visit after 8 weeks of consumption. The possible explanation may involve sensory adaptation [49]. The improvements in neuronal synchronization and myelination observed in subjects who consumed the tuna-oil-containing functional beverage—which is rich in DHA—at a dose of 6000 mg/day corresponded to an increase in serum DHA levels in this group of volunteers. Due to the reputation of DHA with respect to the positive modulation effect on neurogenesis, neurotransmission, myelination, signal transduction, and neuroplasticity [50], we suggest that the beneficial effects relative to cognitive and attention enhancement, together with the improvements in working memory, may be associated with DHA levels in the tuna-oil-containing functional beverage.

Menopausal women also exhibited an increase in oxidative stress with respect to increasing MDA levels, but they exhibited a decrease in SOD [51] and glutathione peroxidase [52]. Inflammatory statuses also increased during the menopausal period. It has been shown that the plasma levels of TNF-α and IL-6 in menopausal women increase [53]. These changes are proposed to be associated with a decrease in cognition during menopause [12,54]. Oxidative stress and inflammation are interrelated because they can promote one another. The elevation of the mentioned substances can trigger tissue damage, including neurons and glial cells inside the brain, resulting in disturbances with respect to neurotransmitters and demyelination [55]. The disturbance of neurotransmitters—such as acetylcholine (ACh), monoamine transmitters, and gamma-aminobutyric acid (GABA)—is also associated with a decrease in cognitive function during menopause and estrogen deprivation. It has been demonstrated that serum estradiol exhibits a negative relationship with acetylcholinesterase (AChE), monoamine oxidase (MAO), and gamma-aminobutyric acid transaminase (GABA-T), which in turn can improve the functions of cholinergic, monoaminergic, and GABAergic transmission, resulting in improvements in selective attention and working memory processes [56,57,58,59]. Our data also reveal that subjects who consumed the tuna-oil-containing functional beverage at a dose of 6000 mg/day also exhibited a reduction in AChE, MAO, GABA-T, MDA, IL-6, and TNF-α and an increase in DHA levels in their serum. Because DHA can improve neurotransmission [50] and decrease oxidative stress and inflammation [60], it is possible that DHA levels present in tuna-oil-containing functional beverages may directly improve neurotransmission, giving rise to improvements in attention, cognition, and working memory. They may also indirectly improve the mentioned parameters either via improvements in oxidative stress statuses and inflammation or via improvements in estrogen synthesis. Although the ovary stops producing estrogen, the adrenal cortex can produce a small amount of estrogen [61]. However, our unpublished data reveal that there were no significant changes in estradiol levels throughout the study period. Therefore, the cognition-enhancing effect of the developed functional beverage is less likely to be a result of the modulation effect of the beverage on estradiol.

Taking all these data together, a high dose of the tuna-oil-containing functional beverage, which is rich in DHA content, may decrease oxidative stress [62] and inflammation [63], resulting in reductions with respect to neurodegeneration and the destruction of brain tissue and myelin sheath in white matter. The aforementioned improvements result in an increase in surviving neurons in selective attention and cognitive processing networks, resulting in improvements with respect to the following: improved cognition manifested by further improvements in the amplitudes of both N100 and P300; reductions in P300 latency; and improvements in performances with respect to the word recognition, simple reaction time, and spatial memory tests. The reduction in neurodegeneration also gives rise to improvements in the dynamic balance of neurotransmitters such as cholinergic, monoaminergic, and GABAergic systems, resulting in cognitive improvements. However, our findings suggest that the effect of low doses of the developed functional beverage may be partly associated with a decrease in telomere length shortening [64]. It has been proposed that longer telomere lengths are associated with a larger total brain and hippocampus volume, giving rise to an increase in connectivity within the brain and resulting in attentional improvements [65]. Unfortunately, there is no measurement for the total brain and hippocampus volume in this study due to budget limitations. This appears to be the weak point of this study. However, the strength of this study is that cognitive functions, including attention and working memory, were measured using both psychometric tests and brain waves. The changes with respect to both data types confirm each other. Furthermore, the possible active ingredient and underlying mechanism were also determined, and all changes provide evidence of improvements in cognitive function. In addition, confounding factors with respect to physical activity and the consumption of food are shown in Supplementary Materials Files S2 and S3. There were no significant changes in the mentioned parameters that corresponded to improvements in cognitive function, attention, and working memory. Thus, improvements in the mentioned parameters are most likely to be associated with the intervention or consumption of the tuna-oil-containing functional beverage. However, further investigations—with more subjects and participants of all genders—are required in order to provide a better understanding regarding the beneficial effect of the developed functional beverage and its precise mechanisms.

Interestingly, subjects who consumed the tuna-oil-containing functional beverage at a dose of 2600 mg/day exhibited improvements with respect to cellular senescence biomarkers, such as telomere length and SIRT1. Although oxidative stress and inflammation can also accelerate telomere shortening [66], no relationships between changes in the mentioned parameters and alterations in the telomere length were observed. It has been reported that the telomerase enzyme plays a crucial role in maintaining the telomere length [67], and this enzyme’s activity can be modified using omega-3, including DHA and EPA [68]. However, the tuna-oil-containing functional beverage at a dose of 6000 mg/day exhibits more EPA and DHA levels, as shown in Supplementary Materials File S4, but no significant increases in telomere length are observed. Therefore, the total concentrations of DHA and EPA may not be a priority factor for maintaining telomere length. Interestingly, it has been reported that the DHA/EPA ratio also plays a crucial role in producing many beneficial effects [69]; for example, the ratios of 1:1 and 2:1 are good for promoting nerve growth factor (NGF) [70], whereas the best ratio for anti-inflammation is 1:1 [69,70]. The improvements in telomere length may possibly be a result of the different ratios of DHA/EPA used in the functional beverage containing both low and high tuna oil amounts (1:1 and 4:1).

The present result also demonstrates an increase in sirtuin 1 (SIRT1) or silent-mating-type information regulation 2 homolog. SIRT1 is an NAD^+^ (nicotinamide adenine dinucleotide)-dependent deacetylase, and it is crucial for the cellular response to diets. It shows very promising properties with respect to extending lifespans [71] and improving brain synaptic plasticity [72]. The increase in synaptic plasticity in turn increases cognitive performance [73]. Moreover, SIRT1 also decreases oxidative stress [74], which can improve cognition, as mentioned earlier. Based on this information, we suggest that beyond reductions in the shortening process of telomeres, a low dose of tuna-oil-containing functional beverages also improves cognition via an increase in SIRT1.

The effect of the tuna-oil-containing functional beverages on SIRT1 is also observed only in subjects who consumed the beverage at a dose of 2600 mg/day. A possible explanation for this phenomenon may be because the DHA/EPA ratio is suitable for this dose, as observed with respect to the changes observed in telomere assessments.

This study also has some limitations. First, it utilized only a small sample size. Moreover, the use of the MDA parameter alone may not be sufficient as a representative indicator of oxidative stress. Validity should be increased via the exploration of more indicators, such as F2-isoprostanes (F2-IsoPs), protein-bound 4-hydroxynonenal (HNE), and 8-hydroxy-2′-deoxyguanosine (8-OHdG); these can indicate the changes induced via oxidative stress relative to various structures, including lipids, proteins, and DNA. Therefore, these points will be considered and investigated in future studies.

## 5. Conclusions

Our results suggest the potential of a functional beverage containing DHA-enriched tuna oil with respect to improving the cognition of perimenopausal and postmenopausal women. The mechanism responsible may depend on the dose of the functional beverage. A high dose of the functioning beverage produced the best effect with respect to improvements in cognitive function, attention, and working memory. The mechanisms may involve improvements in oxidative stress; inflammation; and the suppression of AChE, MAO, and GABA-T, which may improve neurotransmission in attention and cognition circuits. These beneficial effects may be partly associated with the concentrations of DHA. The low-dose functional tuna oil beverage also improved attention and working memory, and the mechanisms responsible may involve the suppression of AChE and an increase in telomere length and SIRT1. The functional beverage at this dose improved many age-related changes, such as telomere length and SIRT1, and may be suitable for antiaging benefits; altogether, these effects may be associated with the ratio of DHA/EPA. The appropriate ratio of DHA/EPA should be 1:1. However, future studies with a larger sample size and examinations into the underlying mechanisms are required to confirm any potential benefits.

## Figures and Tables

**Table 1:** The main ingredients in the placebo and the functional tuna-oil-containing beverage used in this study.

Ingredients	Percent by Weight (mL)		
	Placebo	Functional Beverage at a Dose of 2600 mg/Serving (150 mL)	Functional Beverage at a Dose of 6000 mg/Serving (150 mL)
Active Ingredients			
Soy protein extract	5.000	5.000	5.000
Tuna oil	0.000	1.700	3.900
Sunflower oil	3.900	2.200	0.000
Sesame powder	2.000	2.000	2.000
Total Vitamin B (B1, B2, B3, B5, B6, B7, B9, and B12)	0.020	0.020	0.020
Inactive Ingredients			
Sugar	6.126	6.126	6.126
Water	82.934	82.934	82.934

**Table 2:** The nutritional facts of the placebo and the functional tuna-oil-containing beverage used in this study.

Nutritional Fact per 150 mL	Placebo Containing Sunflower Oil	Tuna-Oil-Containing Beverage (2600 mg/Serving)	Tuna-Oil-Containing Beverage (6000 mg/Serving)
Energy (Kcal)	135	140	140
Protein (g)	7	7	7
Carbohydrate (g)	10	11	10
Sugar	8	8	8
Fat (g)	8	8	8
Omega 3 (mg)	32	860	1941
DHA (mg)	Less than 10	630	1424
EPA (mg)	Less than 10	130	295
Sodium (mg)	128	128	118
Vitamin B1 (mg)	1.43	1.47	1.46
Vitamin B2 (mg)	0.95	0.96	0.94
Niacin (mg NE)	4.96	5.39	5.19
Pantothenic acid (mg)	4.58	4.74	4.47
Vitamin B6 (mg)	1.81	1.85	1.84
Biotin (µg)	66.8	63.4	63.3
Folate (µg)	128	135	124
Vitamin B12 (µg)	1.53	1.63	1.63
Vitamin E (mg alpha-TE)	6.70	6.16	6.01

**Table 3:** Types of primer and sequence of nucleotides of the telomere primer and 36B4 primer.

PCR Primers	Oligomer Sequence (5′-3′)	Amplicon Size
*teloF*	CGGTTTGTTTGGGTTTGGGTTTGGGTTTGGG TTTGGGTT	>76 bp
*teloR*	GGCTTGCCTTACCCTTACCCTTACCC TTACCCTTACCCT	
*36B4F*	CAGCAAGTGGGAAGGTGTAATCC	75 bp
*36B4R*	CCCATTCTATCATCAACGGGTACAA	

**Table 4:** Demographic characteristics of subjects who were assigned to consume the placebo and tuna oil beverages at doses of 2600 and 6000 mg per day (N = 20/arm). Data are presented as mean ± S.D.

**Parameters**	**Baseline**		
	**Placebo** **(n = 20)**	**Functional Beverage at a Dose of 2600 mg/day (n = 20)**	**Functional Beverage at a Dose of 6000 mg/day** **(n = 20)**
Age (year)	49.36 ± 4.25	49.61 ± 4.37 (*p* = 0.884)	49.41 ± 3.78 (*p* = 0.852)
Body temperature (°C)	36.05 ± 0.38	36.15 ± 0.22 (*p* = 0.953)	36.16 ± 0.26 (*p* = 0.685)
Heart rate (beats/min)	70.18 ± 6.24	71.61 ± 7.77 (*p* = 0.640)	70.50 ± 7.99 (*p* = 0.919)
Respiratory rate (breaths/min)	16.18 ± 0.40	16.07 ± 0.28 (*p* = 0.449)	16.33 ± 0.49 (*p* = 0.419)
Systolic blood pressure (mmHg)	119.90 ± 22.66	114.69 ± 9.94 (*p* = 0.954)	111.00 ± 11.82 (*p* = 0.478)
Diastolic blood pressure (mmHg)	67.90 ± 9.01	69.30 ± 6.49 (*p* = 0.670)	63.58 ± 8.31 (*p* = 0.200)
Body weight (kg)	54.71 ± 6.44	54.73 ± 7.12 (*p* = 0.993)	56.44 ± 3.55 (*p* = 0.491)
Body height (cm)	156.63 ± 3.14	156.23 ± 6.26 (*p* = 0.844)	155.91 ± 4.81 (*p* = 0.732)
Body mass index (BMI) (kg/m^2^)	22.26 ± 2.17	22.35 ± 1.79 (*p* = 0.977)	23.22 ± 1.15 (*p* = 0.460)
**Parameters**	**4 weeks**		
	**Placebo** **(n = 20)**	**Functional Beverage at a Dose of 2600 mg/day (n = 20)**	**Functional Beverage at a Dose of 6000 mg/day** **(n = 20)**
Age (year)	49.36 ± 4.25	49.61 ± 4.37 (*p* = 0.884)	49.41 ± 3.78 (*p* = 0.852)
Body temperature (°C)	36.02 ± 0.33	36.17 ± 0.21 (*p* = 0.112)	36.15 ± 0.12 (*p* = 0.100)
Heart rate (beats/min)	72.09 ± 8.68	70.61 ± 8.91 (*p* = 0.681)	70.66 ± 8.45 (*p* = 0.697)
Respiratory rate (breaths/min)	16.27 ± 0.79	16.53 ± 0.78 (*p* = 0.470)	16.66 ± 0.49 (*p* = 0.209)
Systolic blood pressure (mmHg)	112.18 ± 10.57	115.38 ± 10.45 (*p* = 0.454)	110.50 ± 9.92 (*p* = 0.699)
Diastolic blood pressure (mmHg)	69.63 ± 8.37	69.69 ± 5.25 (*p* = 0.985)	66.16 ± 7.86 (*p* = 0.257)
Body weight (kg)	55.36 ± 7.28	54.55 ± 7.59 (*p* = 0.769)	55.90 ± 3.76 (*p* = 0.836)
Body height (cm)	156.63 ± 3.14	156.23 ± 6.26 (*p* = 0.845)	156.00 ± 4.86 (*p* = 0.763)
Body mass index (BMI) (kg/m^2^)	22.51 ± 2.52	22.26 ± 1.86 (*p* = 0.755)	22.97 ± 1.19 (*p* = 0.572)
**Parameters**	**8 weeks**		
	**Placebo** **(n = 20)**	**Functional Beverage at a Dose of 2600 mg/day (n = 20)**	**Functional Beverage at a Dose of 6000 mg/day** **(n = 20)**
Age (year)	49.36 ± 4.25	49.61 ± 4.37 (*p* = 0.884)	49.41 ± 3.78 (*p* = 0.852)
Body temperature (°C)	35.90 ± 0.37	36.19 ± 0.26 (*p* = 0.213)	36.02 ± 0.29 (*p* = 0.521)
Heart rate (beats/min)	72.72 ± 10.04	72.07 ± 8.40 (*p* = 0.864)	72.88 ± 9.29 (*p* = 0.978)
Respiratory rate (breaths/min)	16.54 ± 0.69	16.46 ± 0.66 (*p* = 0.974)	16.75 ± 0.87 (*p* = 0.612)
Systolic blood pressure (mmHg)	106.90 ± 12.19	114.00 ± 11.28 (*p* = 0.132)	109.75 ± 10.17 (*p* = 0.548)
Diastolic blood pressure (mmHg)	70.18 ± 9.72	70.53 ± 5.35 (*p* = 0.910)	67.00 ± 7.51 (*p* = 0.324)
Body weight (kg)	54.92 ± 7.33	54.83 ± 8.03 (*p* = 0.974)	55.45 ± 3.85 (*p* = 0.850)
Body height (cm)	156.63 ± 3.14	156.23 ± 6.26 (*p* = 0.844)	155.91 ± 4.81 (*p* = 0.732)
Body mass index (BMI) (kg/m^2^)	22.35 ± 2.57	22.37 ± 2.05 (*p* = 0.973)	22.81 ± 1.36 (*p* = 0.585)

**Table 5:** Amplitude and latency of N100 and P300 brain waves obtained from the auditory odd ball paradigm of the event-related potential (ERP) of various treated groups, including the placebo and tuna oil beverage groups consuming doses of 2600 and 6000 mg/day (n = 20/arm). Data are expressed as mean ± S.D. *,** *p*-value < 0.05 and 0.01, respectively, compared to the placebo-treated group.

Location	Wave	Treatment Group	Baseline	4 Weeks	8 Weeks
**Fz**	**N100 Latency (ms)**	Placebo	114.18 ± 6.62	111.54 ± 7.45	107.45 ± 5.79
		Functional beverage at a dose of 2600 mg/day	111.61 ± 8.03 (*p* = 0.418)	112.07 ± 9.44 (*p* = 0.882)	107.07 ± 7.83 (*p* = 0.904)
		Functional beverage at a dose of 6000 mg/day	111.83 ± 8.07 (*p* = 0.467)	112.50 ± 8.86 (*p* = 0.794)	108.75 ± 8.71 (*p* = 0.685)
	**N100 Amplitude (µV)**	Placebo	6.98 ± 3.72	6.13 ± 2.64	7.45 ± 3.05
		Functional beverage at a dose of 2600 mg/day	8.92 ± 4.46 (*p* = 0.259)	6.84 ± 2.98 (*p* = 0.258)	7.71 ± 2.78 (*p* = 0.830)
		Functional beverage at a dose of 6000 mg/day	8.17 ± 3.43 (*p* = 0.424)	6.40 ± 3.48 (*p* = 0.460)	7.78 ± 2.98 (*p* = 0.787)
	**P300 Latency (ms)**	Placebo	345.63 ± 12.13	345.90 ± 10.55	346.36 ± 15.40
		Functional beverage at a dose of 2600 mg/day	342.30 ± 13.69 (*p* = 0.542)	343.76 ± 12.97 (*p* = 0.661)	340.61 ± 10.41 (*p* = 0.311)
		Functional beverage at a dose of 6000 mg/day	340.91 ± 13.55 (*p* = 0.397)	344.41 ± 11.54 (*p* = 0.764)	332.00 ± 15.01 * (*p* = 0.017)
	**P300 Amplitude (µV)**	Placebo	21.65 ± 5.22	22.39 ± 4.13	23.44 ± 4.53
		Functional beverage at a dose of 2600 mg/day	25.26 ± 7.81 (*p* = 0.171)	24.84 ± 5.94 (*p* = 0.381)	28.71± 5.35 * (*p* = 0.020)
		Functional beverage at a dose of 6000 mg/day	22.55 ± 5.22 (*p* = 0.736)	29.39 ± 8.60 * (*p* = 0.022)	29.16 ± 4.60 * (*p* = 0.017)
**Cz**	**N100 Latency (ms)**	Placebo	113.81 ± 6.05	113.72 ± 6.71	109.54 ± 7.90
		Functional beverage at a dose of 2600 mg/day	112.00 ± 5.92 (*p* = 0.514)	111.76 ± 8.68 (*p* = 0.563)	108.00 ± 7.40 (*p* = 0.612)
		Functional beverage at a dose of 6000 mg/day	110.33 ± 8.02 (*p* = 0.223)	113.00 ± 8.82 (*p* = 0.833)	111.41 ± 6.82 (*p* = 0.547)
	**N100 Amplitude (µV)**	Placebo	7.28 ± 2.90	5.06 ± 2.01	7.73 ± 3.06
		Functional beverage at a dose of 2600 mg/day	7.78 ± 3.52 (*p* = 0.401)	8.51 ± 2.77 ** (*p* = 0.001)	8.02 ± 3.59 (*p* = 0.827)
		Functional beverage at a dose of 6000 mg/day	7.55 ± 1.42 (*p* = 0.268)	7.20 ± 3.15 * (*p* = 0.029)	7.39 ± 2.91 (*p* = 0.803)
	**P300 Latency (ms)**	Placebo	341.90 ± 12.43	348.00 ± 11.58	346.54 ± 16.74
		Functional beverage at a dose of 2600 mg/day	344.07 ± 10.81 (*p* = 0.653)	347.76 ± 10.10 (*p* = 0.960)	343.53 ± 9.42 (*p* = 0.604)
		Functional beverage at a dose of 6000 mg/day	337.41 ± 11.80 (*p* = 0.362)	346.33 ± 11.95 (*p* = 0.724)	337.41 ± 15.38 (*p* = 0.128)
	**P300 Amplitude (µV)**	Placebo	20.40 ± 4.78	19.65 ± 5.84	22.83 ± 7.24
		Functional beverage at a dose of 2600 mg/day	24.46 ± 9.17 (*p* = 0.118)	24.56 ± 5.77 (*p* = 0.091)	20.67 ± 5.01 (*p* = 0.436)
		Functional beverage at a dose of 6000 mg/day	23.34 ± 6.80 (*p* = 0.235)	26.28 ± 7.03 * (*p* = 0.028)	25.97 ± 6.45 (*p* = 0.260)

**Table 6:** Changes in response times and the response accuracy percentage of subjects who consumed the placebo and tuna-oil-containing functional beverages at doses of 2600 and 6000 mg per day in the computerized battery test comprising various recognition tests—including simple reaction time, choice reaction time, digit vigilance, word recognition, numeric recognition, picture recognition, and spatial recognition tests—prior to consumption and after 4 and 8 weeks of consumption (N = 20/arm). Data are expressed as mean ± S.D. *, ** *p*-value < 0.05 and 0.01, respectively, compared to the placebo-treated group.

Cognitive Domains	Test Items	Treatment Group	Baseline	4 Weeks	8 Weeks
**Word Recognition**	Time	Placebo	1150.43 ± 234.14	1186.94 ± 224.87	1179.63 ± 211.74
		Functional beverage at a dose of 2600 mg/day	1228.28 ± 291.10 (*p* = 0.430)	1129.06 ± 178.61 (*p* = 0.447)	1071.68 ± 145.15 (*p* = 0.107)
		Functional beverage at a dose of 6000 mg/day	1141.21 ± 164.94 (*p* = 0.927)	1026.17 ± 128.86 * (*p* = 0.046)	1024.64 ± 111.11 * (*p* = 0.026)
	%Accuracy	Placebo	87.87 ± 7.64	85.75 ± 8.18	90.00 ± 8.69
		Functional beverage at a dose of 2600 mg/day	84.87 ± 10.60 (*p* = 0.465)	89.48 ± 8.15 (*p* = 0.319)	86.66 ± 9.91 (*p* = 0.980)
		Functional beverage at a dose of 6000 mg/day	92.22 ± 6.41 (*p* = 0.225)	93.33 ± 4.71 * (*p* = 0.020)	90.00 ± 9.21 (*p* = 0.925)
**Picture Recognition**	Time	Placebo	1227.98 ± 203.11	1232.52 ± 167.04	1219.79 ± 212.98
		Functional beverage at a dose of 2600 mg/day	1326.06 ± 352.77 (*p* = 0.543)	1180.59 ± 197.54 (*p* = 0.480)	1206.40 ± 260.07 (*p* = 0.882)
		Functional beverage at a dose of 6000 mg/day	1246.29 ± 201.84 (*p* = 0.854)	1139.84 ± 162.30 (*p* = 0.219)	1113.67 ± 170.02 (*p* = 0.254)
	%Accuracy	Placebo	86.81 ± 8.15	89.09 ± 5.39	88.63 ± 6.74
		Functional beverage at a dose of 2600 mg/day	90.00 ± 10.41 (*p* = 0.289)	88.46 ± 7.18 (*p* = 0.785)	89.23 ± 8.13 (*p* = 0.404)
		Functional beverage at a dose of 6000 mg/day	92.50 ± 5.44 (*p* = 0.091)	92.50 ± 5.84 (*p* = 0.161)	91.25 ± 5.28 (*p* = 0.213)
**Simple Reaction**	Time	Placebo	620.10 ± 114.57	668.66 ± 109.58	654.99 ± 164.52
		Functional beverage at a dose of 2600 mg/day	649.34 ± 117.93 (*p* = 0.530)	593.40 ± 70.39 * (*p* = 0.035)	624.16 ± 100.14 (*p* = 0.581)
		Functional beverage at a dose of 6000 mg/day	641.42 ± 104.44 (*p* = 0.653)	568.79 ± 57.36 ** (*p* = 0.007)	631.08 ± 137.80 (*p* = 0.674)
**Digit Vigilance**	Time	Placebo	631.92 ± 46.53	662.61 ± 57.83	642.35 ± 53.98
		Functional beverage at a dose of 2600 mg/day	620.92 ± 56.16 (*p* = 0.931)	644.25 ± 52.91 (*p* = 0.401)	642.32 ± 39.41 (*p* = 0.839)
		Functional beverage at a dose of 6000 mg/day	657.18 ± 40.21 (*p* = 0.140)	641.63 ± 47.06 (*p* = 0.346)	649.28 ± 46.23 (*p* = 0.498)
	%Accuracy	Placebo	95.10 ± 5.49	94.05 ± 5.99	95.22 ± 5.53
		Functional beverage at a dose of 2600 mg/day	96.25 ± 3.83 (*p* = 0.765)	95.85 ± 5.00 (*p* = 0.374)	95.06 ± 4.28 (*p* = 0.598)
		Functional beverage at a dose of 6000 mg/day	95.62 ± 3.81 (*p* = 0.950)	95.51 ± 2.81 (*p* = 0.876)	94.65 ± 4.30 (*p* = 0.556)
**Choice Reaction Time**	Time	Placebo	800.03 ± 116.80	816.85 ± 104.14	801.82 ± 107.78
		Functional beverage at a dose of 2600 mg/day	812.59 ± 105.03 (*p* = 0.768)	794.97 ± 107.00 (*p* = 0.604)	814.06 ± 72.50 (*p* = 0.354)
		Functional beverage at a dose of 6000 mg/day	799.58 ± 85.96 (*p* = 0.992)	786.04 ± 94.40 (*p* = 0.475)	785.19 ± 89.79 (*p* = 0.865)
	%Accuracy	Placebo	98.54 ± 1.57	97.45 ± 2.02	98.90 ± 1.64
		Functional beverage at a dose of 2600 mg/day	97.38 ± 2.22 (*p* = 0.178)	98.92 ± 1.32 (*p* = 0.052)	98.92 ± 1.75 (*p* = 0.973)
		Functional beverage at a dose of 6000 mg/day	98.00 ± 2.26 (*p* = 0.643)	98.33 ± 2.23 (*p* = 0.177)	97.50 ± 2.28 (*p* = 0.095)
**Spatial Memory**	Time	Placebo	1390.59 ± 314.27	1348.38 ± 253.38	1471.43 ± 235.28
		Functional beverage at a dose of 2600 mg/day	1460.04 ± 410.53 (*p* = 0.582)	1320.48 ± 158.46 (*p* = 0.797)	1272.64 ± 236.83 * (*p* = 0.049)
		Functional beverage at a dose of 6000 mg/day	1226.30 ± 191.46 (*p* = 0.196)	1258.69 ± 349.11 (*p* = 0.420)	1163.61 ± 226.35 ** (*p* = 0.004)
	%Accuracy	Placebo	87.50 ± 7.20	92.17 ± 7.01	91.91 ± 9.66
		Functional beverage at a dose of 2600 mg/day	87.00 ± 15.25 (*p* = 0.921)	92.50 ± 13.15 (*p* = 0.201)	94.23 ± 7.21 (*p* = 0.857)
		Functional beverage at a dose of 6000 mg/day	93.74 ± 10.27 (*p* = 0.223)	95.13 ± 5.70 (*p* = 0.245)	94.44 ± 7.95 (*p* = 0.652)
**Numeric Working Memory**	Time	Placebo	1199.77 ± 295.98	1117.28 ± 152.77	1107.26 ± 212.83
		Functional beverage at a dose of 2600 mg/day	1101.40 ± 126.64 (*p* = 0.258)	1019.02 ± 136.67 (*p* = 0.110)	1005.46 ± 153.27 (*p* = 0.182)
		Functional beverage at a dose of 6000 mg/day	1104.45 ± 182.70 (*p* = 0.282)	1037.64 ± 149.55 (*p* = 0.200)	1013.33 ± 181.12 (*p* = 0.226)
	%Accuracy	Placebo	96.36 ± 6.23	97.57 ± 3.97	95.15 ± 6.03
		Functional beverage at a dose of 2600 mg/day	92.56 ± 10.73 (*p* = 0.319)	92.56 ± 10.11 (*p* = 0.262)	92.82 ± 12.16 (*p* = 0.576)
		Functional beverage at a dose of 6000 mg/day	93.33 ± 9.75 (*p* = 0.443)	94.16 ± 8.89 (*p* = 0.502)	96.11 ± 5.29 (*p* = 0.600)

**Table 7:** Changes in oxidative stress indices—such as malondialdehyde (MDA), superoxide dismutase (SOD), catalase (CAT), and glutathione peroxidase (GPx)—and inflammatory cytokines—including tumor necrosis factor-alpha (TNF-α) and interleukin-6 (IL-6)—in the serum of subjects who consumed the placebo or functional beverage at doses of 2600 and 6000 mg per day at baseline and after 4 and 8 weeks of consumption (N = 20/arm). Data are expressed as mean ± S.D. * *p*-value < 0.05; compared to the placebo-treated group.

Parameters	Treatment Group	Baseline	4 Weeks	8 Weeks
MDA (umol/mg protein)	Placebo	8.45 ± 5.41	7.72 ± 4.43	4.04 ± 1.28
	Functional beverage at a dose of 2600 mg/day	9.04 ± 6.35 (*p* = 0.822)	5.45 ± 2.23 (*p* = 0.088)	5.11 ± 1.47 (*p* = 0.156)
	Functional beverage at a dose of 6000 mg/day	10.64 ± 6.30 (*p* = 0.401)	4.98 ± 1.47 * (*p* = 0.041)	5.76 ±2.46(*p* = 0.385)
SOD (U/mg protein)	Placebo	12.68 ± 4.06	15.46 ± 1.78	18.64 ± 8.21
	Functional beverage at a dose of 2600 mg/day	11.76 ± 2.87 (*p* = 0.740)	12.94 ± 4.11 (*p* = 0.180)	16.04 ± 8.34 (*p* = 0.535)
	Functional beverage at a dose of 6000 mg/day	14.43± 5.66 (*p* = 0.514)	13.74 ± 2.07 (*p* = 0.368)	25.46 ± 1.99 * (*p* = 0.037)
CAT (U/mg protein)	Placebo	23.52 ± 0.83	23.86 ±4.74	21.07 ± 2.39
	Functional beverage at a dose of 2600 mg/day	22.11 ± 1.86 (*p* = 0.127)	24.38 ± 8.05 (*p* = 0.974)	23.69 ± 5.46 (*p* = 0.388)
	Functional beverage at a dose of 6000 mg/day	22.46 ± 1.92(*p* = 0.304)	21.92 ± 2.88 (*p* = 0.703)	24.24 ± 5.64 (*p* = 0.269)
GPx. (U/mg protein)	Placebo	0.09 ± 0.07	0.07 ± 0.05	0.17 ± 0.02
	Functional beverage at a dose of 2600 mg/day	0.08 ± 0.05 (*p* = 0.671)	0.07 ± 0.05 (*p* = 0.938)	0.18 ± 0.12 (*p* = 0.937)
	Functional beverage at a dose of 6000 mg/day	0.10 ± 0.06 (*p* = 0.793)	0.13 ± 0.08 * (*p* = 0.042)	0.18 ± 0.07 (*p* = 0.839)
TNF-α (ng/mL)	Placebo	1.13 ± 0.82	0.83 ± 0.57	1.23 ± 0.72
	Functional beverage at a dose of 2600 mg/day	0.97 ± 0.38 (*p* = 0.599)	0.99 ± 0.50 (*p* = 0.469)	0.93 ± 0.59 (*p* = 0.281)
	Functional beverage at a dose of 6000 mg/day	0.92 ± 0.44 (*p* = 0.509)	0.52 ± 0.26 * (*p* = 0.044)	0.58 ± 0.24 * (*p* = 0.029)
IL-6 (pg/mL)	Placebo	31.40 ± 4.59	36.64 ± 4.08	38.04 ± 5.90
	Functional beverage at a dose of 2600 mg/day	31.65 ± 6.88 (*p* = 0.975)	29.96 ± 4.28 (*p* = 0.380)	29.28 ± 4.42 (*p* = 0.276)
	Functional beverage at a dose of 6000 mg/day	24.77 ± 5.21 (*p* = 0.447)	26.22 ± 6.71 (*p* = 0.202)	24.28 ± 4.62 * (*p* = 0.046)

## Data Availability

The data presented in this study are available upon request from the corresponding author.

## Supplementary Materials

The following supporting information can be downloaded at https://www.mdpi.com/article/10.3390/antiox14050520/s1. Fingerprint chromatograms of lipids are provided in S1, and the physical, activity, food consumption, and nutritional profiles of tuna-oil-containing beverages and the placebo are provided in S2, S3, and S4, respectively. Moreover, the content regarding genes used in this study was provided in S5.

## Glossary

N100: Negative 100; P300: Positive 300; MDA: malondialdehyde; SOD: superoxide dismutase; CAT: catalase; GPx: glutathione peroxidase; TNF-α: tumor necrosis factor-alpha; IL-6: interleukin-6; eNOS: endothelial nitric oxide synthase; AChE: acetylcholinesterase; MAO: monoamine oxidase; GABA-T: gamma-amino-butyric acid transaminase; SIRT1: sirtuin-1; NAD^+^: nicotinamide adenine dinucleotide; HRT: hormone replacement therapy; QoL: quality of life; PUFAs: polyunsaturated fatty acids; LC-n3-FA: long-chain polyunsaturated omega-3 fatty acids; DHA: docosahexaenoic acid; EPA: eicosapentaenoic acid; ERP: event-related potential; EEG: electroencephalogram; Ag: silver; Ag-Xl: silver chloride; Hz: hertz; dB: decibel; VGA: Video Graphic Adapter; PCR: polymerase chain reaction; LC–MS/MS: liquid chromatography–tandem mass spectrometry; HPLC: high-performance liquid chromatography; ESI: electrospray ionization; F2-IsoPs: F2-isoprostanes; HNE: 4-hydroxynonenal; 8-OHdG: 8-Hydroxy-2′-deoxyguanosine.
