# Supplementary material for: A Randomized, Double-Blind, Placebo-Controlled, Parallel-Group, 8-Week Pilot Study of Tuna-Byproduct-Derived Novel Supplements for Managing Cellular Senescence and Cognitive Decline in Perimenopausal and Postmenopausal Women
Source: Antioxidants (Basel). 2025 Apr 27;14(5):520. doi: 10.3390/antiox14050520 (PMC12108292; doi:10.3390/antiox14050520)
Supplement: Supplementary file 1 [file antioxidants-14-00520-s001.zip › S2 Physical activity.pdf]

Table S1 Physical activity of adulthood volunteers who consumed placebo, or the functional drink at the doses of 2600, and 6000 mg per day at baseline. (N=20/arm) Data are expressed as mean±S.E.M.

| **Parameter** | | **Baseline** | | |
|---|---|---|---|---|
| | | Placebo | Functional drink at the dose of 2,600 mg/day | Functional drink at the dose of 6,000 mg/day |
| high intensity activity | Run fast | 0.45 ± 0.36 | 0.15 ± 0.15 (p=0.450) | 0.00 ± 0.00 (p=0.131) |
| | Lifting heavy objects | 3.36 ± 0.97 | 1.76 ± 0.71 (p=0.270) | 3.25 ± 0.99 (p= 0.845) |
| | Exercise (m) | 14.27 ± 9.29 | 53.69 ± 20.46 (p=0.059) | 97.91 ± 40.00 (p=0.219) |
| Moderate intensity activity | Cycling on flat ground | 2.27 ± 0.90 | 0.76 ± 0.56 (p=0.129) | 0.66 ± 0.51 (p=0.112) |
| | Lift light weights | 2.89 ± 1.09 | 1.15 ± 0.72 (p=0.128) | 2.91 ± 1.04 (p=0.843) |
| Walking | walk fast | 2.63 ± 1.04 | 2.76 ± 0.85 (p=0.663) | 2.16 ± 0.93 (p=0.569) |
| | Walk slowly | 6.81 ± 0.18 | 4.61 ± 0.81 *(p=0.019) | 3.66 ± 1.01 *(p=0.022) |
| | Walk at home | 5.81 ± 0.79 | 4.30 ± 0.87 (p=0.101) | 5.08 ± 0.89 (p=0.401) |
| | Travel to various places | 4.81 ± 0.91 | 3.38 ± 0.87 (p=0.325) | 5.25 ± 0.86 (p=0.623) |
| | Walk for relaxation | 2.72 ± 1.03 | 4.15 ± 0.95 (p=0.465) | 2.50 ± 0.97 (p=0.862) |
| | Sit at the desk | 2.72 ± 1.00 | 4.61 ± 0.86 (p=0.137) | 3.16 ± 0.97 (p=0.787) |
| Sitting | Sit and watch TV | 5.18 ± 0.77 | 3.46 ± 0.89 (p=0.163) | 3.08 ± 0.98 (p=0.117) |
| | Sit and chat with friends | 3.45 ± 1.03 | 3.30 ± 0.90 (p=0.877) | 3.08 ± 0.82 (p=0.898) |
| | Sit and relax | 5.27 ± 0.79 | 3.61 ± 0.91 (p=0.160) | 4.83 ± 0.77 (p=0.527) |
| | Sit and read a book | 2.00 ± 0.97 | 1.61 ± 0.72 (p=0.947) | 0.33 ± 0.25 (p=0.126) |
| Other activities | | 0.00 ± 0.00 | 0.00 ± 0.00 | 0.00 ± 0.00 |

Table S2 Physical activity of adulthood volunteers who consumed placebo, or the functional drink at the doses of 2600, and 6000 mg per day after 4-week of consumption. (N=20/arm) Data are expressed as mean±S.E.M.

| **Parameter** | | **4-week** | | |
|---|---|---|---|---|
| | | Placebo | Functional drink at the dose of 2,600 mg/day | Functional drink at the dose of 6,000 mg/day |
| high intensity activity | Run fast | 0.72 ± 0.40 | 0.61 ± 0.47 (p=0.540) | 0.00 ± 0.00 (p=0.058) |
| | Lifting heavy objects | 3.33 ± 1.01 | 1.92 ± 0.83 (p=0.145) | 1.50 ± 0.81 (p=0.058) |
| | Exercise (m) | 30.45 ± 16.01 | 58.76 ± 34.12 (p=0.674) | 71.66 ± 25.04 (p=0.207) |
| | Cycling on flat ground | 2.81 ± 0.92 | 0.92 ± 0.57 (p=0.052) | 1.33 ± 0.74 (p=0.095) |
| Moderate intensity activity | Lift light weights | 2.09 ± 0.95 | 1.69 ± 0.84 (p=0.544) | 2.25 ± 0.92 (p=0.918) |
| | walk fast | 3.72 ± 1.00 | 2.61 ± 0.90 (p=0.397) | 3.75 ± 0.98 (p=0.921) |
| | Walk slowly | 5.63 ± 0.84 | 5.38 ± 0.75 (p=0.856) | 5.50 ± 0.75 (p=0.850) |
| Walking | Walk at home | 5.81 ± 0.73 | 4.76 ± 1.30 (p=0.238) | 4.58 ± 0.90 (p=0.404) |
| | Travel to various places | 5.27 ± 0.89 | 3.07 ± 0.90 (p=0.052) | 3.50 ± 0.94 (p=0.150) |
| | Walk for relaxation | 3.81 ± 0.97 | 3.69 ± 0.89 (p=0.831) | 3.83 ± 0.98 (p=0.974) |
| | Sit at the desk | 2.90 ± 0.94 | 3.00 ± 0.82 (p=0.810) | 1.58 ± 0.83 (p=0.232) |
| | Sit and watch TV | 4.63 ± 1.00 | 3.46 ± 0.88 (p=0.250) | 5.33 ± 0.79 (p=0.716) |
| Sitting | Sit and chat with friends | 4.54 ± 1.03 | 4.30 ± 0.88 (p=0.586) | 3.91 ± 0.96 (p=0.628) |
| | Sit and relax | 5.63 ± 0.71 | 4.30 ± 0.88 (p=0.243) | 5.00 ± 0.90 (p=0.596) |
| | Sit and read a book | 1.45 ± 0.69 | 1.00 ± 0.59 (p=0.516) | 1.16 ± 0.78 (p=0.424) |
| Other activities | | 0.00 ± 0.00 | 0.00 ± 0.00 | 0.00 ± 0.00 |

Table S3 Physical activity of adulthood volunteers who consumed placebo, or the functional drink at the doses of 2600, and 6000 mg per day after 8-week of consumption. (N=20/arm) Data are expressed as mean±S.E.M.

| **Parameter** | | **8-week** | | |
|---|---|---|---|---|
| | | Placebo | Functional drink at the dose of 2,600 mg/day | Functional drink at the dose of 6,000 mg/day |
| high intensity activity | Run fast | 0.18 ± 0.18 | 0.46 ± 0.24 (p=0.236) | 0.00 ± 0.00 (p=0.296) |
| | Lifting heavy objects | 3.63 ± 1.01 | 2.00 ± 0.80 (p=0.282) | 2.41 ± 0.81 (p=0.361) |
| | Exercise (m) | 24.54 ± 15.09 | 79.23 ± 28.23 (p=0.122) | 36.25 ± 19.82 (p=0.650) |
| | Cycling on flat ground | 2.56 ± 0.97 | 0.85 ± 0.39 (p=0.143) | 0.92 ± 0.54 (p=0.122) |
| Moderate intensity activity | Lift light weights | 3.54 ± 1.03 | 1.61 ± 0.85 (p=0.085) | 2.66 ± 0.95 (p=0.506) |
| | walk fast | 3.81 ± 0.99 | 2.69 ± 0.78 (p=0.429) | 2.16 ± 0.88 (p=0.229) |
| | Walk slowly | 5.90 ± 0.69 | 3.92 ± 0.88 (p=0.135) | 5.83 ± 0.66 (p=0.936) |
| Walking | Walk at home | 5.72 ± 0.85 | 4.38 ± 0.95 (p=0.324) | 4.25 ± 0.89 (p=0.190) |
| | Travel to various places | 4.63 ± 1.00 | 3.15 ± 0.93 (p=0.264) | 3.83 ± 0.97 (p=0.580) |
| | Walk for relaxation | 3.18 ± 1.10 | 2.46 ± 0.90 (p=0.769) | 1.91 ± 0.75 (p=0.636) |
| | Sit at the desk | 2.36 ± 0.94 | 2.46 ± 0.92 (p=0.922) | 2.08 ± 0.81 (p=0.733) |
| | Sit and watch TV | 3.81 ± 1.10 | 2.38 ± 0.89 (p=0.254) | 3.91 ± 0.89 (p=0.891) |
| Sitting | Sit and chat with friends | 4.63 ± 1.00 | 3.00 ± 0.86 (p=0.255) | 2.66 ± 0.87 (p=0.158) |
| | Sit and relax | 6.22 ± 0.52 | 4.27 ± 0.92 (p=0.073) | 5.25 ± 0.78 (p=0.422) |
| | Sit and read a book | 1.09 ± 0.66 | 1.07 ± 0.54 (p=0.755) | 1.33 ± 0.78 (p=0.968) |
| Other activities | | 0.00 ± 0.00 | 0.00 ± 0.00 | 0.00 ± 0.00 |
