# Supplementary material for: A Randomized, Double-Blind, Placebo-Controlled, Parallel-Group, 8-Week Pilot Study of Tuna-Byproduct-Derived Novel Supplements for Managing Cellular Senescence and Cognitive Decline in Perimenopausal and Postmenopausal Women
Source: Antioxidants (Basel). 2025 Apr 27;14(5):520. doi: 10.3390/antiox14050520 (PMC12108292; doi:10.3390/antiox14050520)
Supplement: Supplementary file 1 [file antioxidants-14-00520-s001.zip › S1 Fatty acids chromatogram of Tuna oil-containing beverage_19042025 JW.pdf]

File S1 Fatty acids chromatogram of tuna oil-containing beverage in clinical study

Clinical study - Start Intervention

SGS Test Report no.	**Sample:** 1 Formula 11 (Placebo) at <u>start intervention</u>, Sunflower oil-containing beverage in clinical study
5239167	

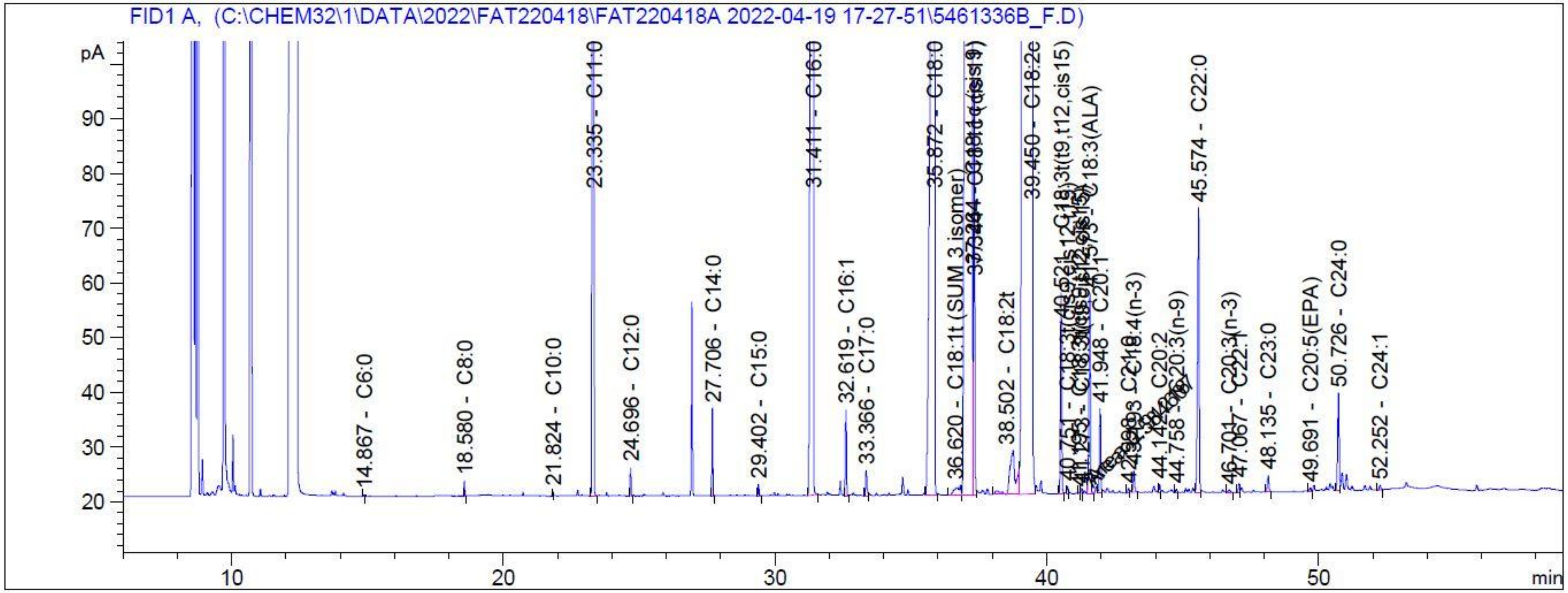

Total Fatty acid	mg/100ml
Total fatty acid	5058.70
Butyric acid (C4:0)	Less than 10.00
Caproic acid (C6:0)	Less than 10.00
Caprylic acid (C8:0)	Less than 10.00
Capric acid (C10:0)	Less than 10.00
Lauric acid (C12:0)	Less than 10.00
Tridecanoic acid (C13:0)	Less than 10.00
Myristic acid (C14:0)	Less than 10.00
Myristoleic acid (C14:1)	Less than 10.00
Pentadecanoic acid (C15:0)	Less than 10.00
cis-10-Pentadecenoic acid (C15:1)	Less than 10.00
Palmitic acid (C16:0)	470.26
trans-9-Hexadecenoic acid (C16:1t)	Less than 10.00
Palmitoleic acid (C16:1)	Less than 10.00

Heptadecanoic acid (C17:0)	Less than 10.00
cis-10-Heptadecenoic acid (C17:1)	Less than 10.00
Stearic acid (C18:0)	264.23
C18:1t (Sum3 isomer)	Less than 10.00
cis-9-Oleic acid (C18:1 c)	1371.30
cis-11-vacenic acid (C18:1c)	37.39
cis-12-octadecenoic acid (C18:1c)	Less than 10.00
trans-9,12-Octadecadienoic acid (C18:2t)	18.10
cis-9,12-Octadecadienoic acid (C18:2 c)	2816.26
18:3 trans-9, trans-12, trans-15-octadecatrienoic	Less than 10.00
Arachidic acid (C20:0)	18.44
18:3 trans-9, trans-12, cis-15-octadecatrienoic	Less than 10.00
18:3 trans-9, cis-12, trans-15-octadecatrienoic	Less than 10.00
Gamma-Linolenic acid (C18:3 GLA)	Less than 10.00
18:3 cis-9, trans-12, trans-15-octadecatrienoic	Less than 10.00
18:3 cis-9, cis-12, trans-15-octadecatrienoic	Less than 10.00
18:3 cis-9, trans-12, cis-15-octadecatrienoic	Less than 10.00
18:3 trans-9, cis-12, cis-15-octadecatrienoic	Less than 10.00
trans-11-Eicosenoic acid (C20:1t)	Less than 10.00
alpha-Linolenic acid (C18:3 ALA)	19.33
cis-11-Eicosenoic acid (C20:1)	Less than 10.00
Heneicosanoic acid (C21:0)	Less than 10.00
Stearidonic acid (18:4)	Less than 10.00
cis-11,14-Eicosadienoic acid (C20:2)	Less than 10.00
cis-5,8,11-Eicosatrienoic acid (C20:3 n-9)	Less than 10.00
Behenic acid (C22:0)	31.86
cis-8,11,14-Eicosatrienoic acid (C20:3 n-6)	Less than 10.00
trans-13-Docosenoic acid (C22:1t)	Less than 10.00
cis-11,14,17-Eicosatrienoic acid (C20:3 n-3)	Less than 10.00
cis-5,8,11,14-Eicosatetraenoic acid (C20:4 ARA)	Less than 10.00
Erucic acid (C22:1)	Less than 10.00
Tricosanoic acid (C23:0)	Less than 10.00
cis 8,11,14,17 Eicosatetraenoic acid C20:4(n-3)	Less than 10.00
cis-13,16-Docosadienoic acid (C22:2)	Less than 10.00
cis-5,8,11,14,17-Eicosapentaenoic acid (C20:5 EPA)	Less than 10.00
Lignoceric acid (C24:0)	11.53
cis-13,16,19-Docosatrienoic acid (C22:3)	Less than 10.00
Nervonic acid (C24:1)	Less than 10.00
Adrenic acid (C22:4)	Less than 10.00
Docosapentaenoic acid (C22:5)	Less than 10.00
cis-7,10,13,16,19-Docosapentaenoic acid (C22:5 DPA)	Less than 10.00
cis-4,7,10,13,16,19-Docosahexaenoic acid (C22:6 DHA)	Less than 10.00

Clinical study - Start Intervention

SGS Test Report no.	Sample: 2 Formula 22 at start intervention, 2600 mg Tuna oil-containing beverage in clinical study
5239166	

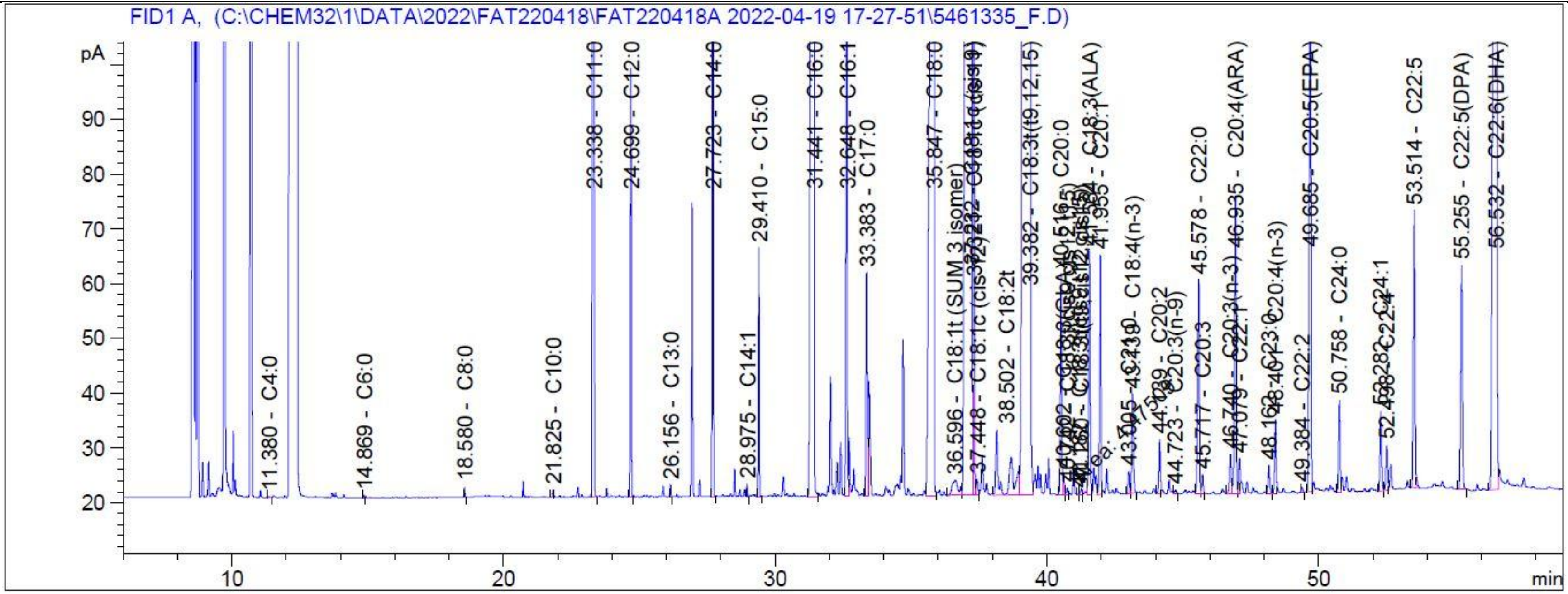

Total Fatty acid	mg/100ml
Total fatty acid	4754.41
Butyric acid (C4:0)	Less than 10.00
Caproic acid (C6:0)	Less than 10.00
Caprylic acid (C8:0)	Less than 10.00
Capric acid (C10:0)	Less than 10.00
Lauric acid (C12:0)	24.84
Tridecanoic acid (C13:0)	Less than 10.00
Myristic acid (C14:0)	49.76
Myristoleic acid (C14:1)	Less than 10.00
Pentadecanoic acid (C15:0)	15.24
cis-10-Pentadecenoic acid (C15:1)	Less than 10.00
Palmitic acid (C16:0)	645.82
trans-9-Hexadecenoic acid (C16:1t)	Less than 10.00
Palmitoleic acid (C16:1)	64.28
Heptadecanoic acid (C17:0)	19.01
cis-10-Heptadecenoic acid (C17:1)	Less than 10.00
Stearic acid (C18:0)	289.05

SGS Test Report no.	Sample: 2 Formula 22 at start intervention, 2600 mg Tuna oil-containing beverage in clinical study

C18:1t (Sum3 isomer)	Less than 10.00
cis-9-Oleic acid (C18:1 c)	1089.80
cis-11-vacenic acid (C18:1c)	58.48
cis-12-octadecenoic acid (C18:1c)	Less than 10.00
trans-9,12-Octadecadienoic acid (C18:2t)	23.01
cis-9,12-Octadecadienoic acid (C18:2 c)	1815.29
18:3 trans-9, trans-12, trans-15-octadecatrienoic	Less than 10.00
Arachidic acid (C20:0)	19.53
18:3 trans-9, trans-12, cis-15-octadecatrienoic	Less than 10.00
18:3 trans-9, cis-12, trans-15-octadecatrienoic	Less than 10.00
Gamma-Linolenic acid (C18:3 GLA)	Less than 10.00
18:3 cis-9, trans-12, trans-15-octadecatrienoic	Less than 10.00
18:3 cis-9, cis-12, trans-15-octadecatrienoic	Less than 10.00
18:3 cis-9, trans-12, cis-15-octadecatrienoic	Less than 10.00
18:3 trans-9, cis-12, cis-15-octadecatrienoic	Less than 10.00
trans-11-Eicosenoic acid (C20:1t)	Less than 10.00
alpha-Linolenic acid (C18:3 ALA)	22.65
cis-11-Eicosenoic acid (C20:1)	21.97
Heneicosanoic acid (C21:0)	Less than 10.00
Stearidonic acid (18:4)	10.66
cis-11,14-Eicosadienoic acid (C20:2)	Less than 10.00
cis-5,8,11-Eicosatrienoic acid (C20:3 n-9)	Less than 10.00
Behenic acid (C22:0)	22.22
cis-8,11,14-Eicosatrienoic acid (C20:3 n-6)	Less than 10.00
trans-13-Docosenoic acid (C22:1t)	Less than 10.00
cis-11,14,17-Eicosatrienoic acid (C20:3 n-3)	Less than 10.00
cis-5,8,11,14-Eicosatetraenoic acid (C20:4 ARA)	31.47
Erucic acid (C22:1)	Less than 10.00
Tricosanoic acid (C23:0)	Less than 10.00
cis 8,11,14,17 Eicosatetraenoic acid C20:4(n-3)	Less than 10.00
cis-13,16-Docosadienoic acid (C22:2)	Less than 10.00
cis-5,8,11,14,17-Eicosapentaenoic acid (C20:5 EPA)	79.30
Lignoceric acid (C24:0)	10.40
cis-13,16,19-Docosatrienoic acid (C22:3)	Less than 10.00
Nervonic acid (C24:1)	Less than 10.00
Adrenic acid (C22:4)	Less than 10.00
Docosapentaenoic acid (C22:5)	30.43
cis-7,10,13,16,19-Docosapentaenoic acid (C22:5 DPA)	28.76
cis-4,7,10,13,16,19-Docosahexaenoic acid (C22:6 DHA)	382.44

Clinical study - Start Intervention

SGS Test Report no.	Sample: 3 Formula 33 at start intervention, 6000 mg Tuna oil-containing beverage in clinical study
5239160	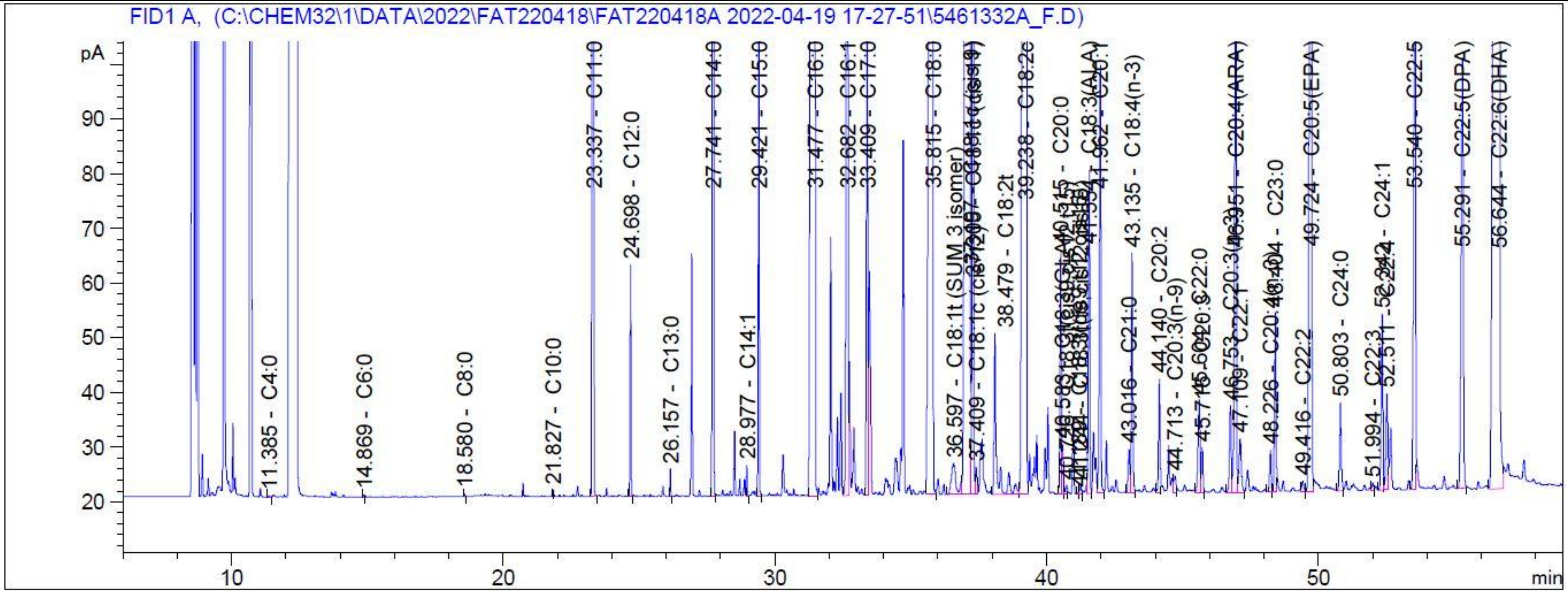

Total Fatty acid	mg/100ml
Total fatty acid	4843.21
Butyric acid (C4:0)	Less than 10.00
Caproic acid (C6:0)	Less than 10.00
Caprylic acid (C8:0)	Less than 10.00
Capric acid (C10:0)	Less than 10.00
Lauric acid (C12:0)	13.77
Tridecanoic acid (C13:0)	Less than 10.00
Myristic acid (C14:0)	112.97
Myristoleic acid (C14:1)	Less than 10.00
Pentadecanoic acid (C15:0)	35.86
cis-10-Pentadecenoic acid (C15:1)	Less than 10.00
Palmitic acid (C16:0)	926.94
trans-9-Hexadecenoic acid (C16:1t)	Less than 10.00
Palmitoleic acid (C16:1)	147.74
Heptadecanoic acid (C17:0)	46.95
cis-10-Heptadecenoic acid (C17:1)	Less than 10.00
Stearic acid (C18:0)	344.92
C18:1t (Sum3 isomer)	Less than 10.00

SGS Test Report no.	Sample: 3 Formula 33 at start intervention, 6000 mg Tuna oil-containing beverage in clinical study

cis-9-Oleic acid (C18:1 c)	852.28
cis-11-vacenic acid (C18:1c)	90.41
cis-12-octadecenoic acid (C18:1c)	Less than 10.00
trans-9,12-Octadecadienoic acid (C18:2t)	27.36
cis-9,12-Octadecadienoic acid (C18:2 c)	662.32
18:3 trans-9, trans-12, trans-15-octadecatrienoic	Less than 10.00
Arachidic acid (C20:0)	23.53
18:3 trans-9, trans-12, cis-15-octadecatrienoic	Less than 10.00
18:3 trans-9, cis-12, trans-15-octadecatrienoic	Less than 10.00
Gamma-Linolenic acid (C18:3 GLA)	Less than 10.00
18:3 cis-9, trans-12, trans-15-octadecatrienoic	Less than 10.00
18:3 cis-9, cis-12, trans-15-octadecatrienoic	Less than 10.00
18:3 cis-9, trans-12, cis-15-octadecatrienoic	Less than 10.00
18:3 trans-9, cis-12, cis-15-octadecatrienoic	Less than 10.00
trans-11-Eicosenoic acid (C20:1t)	Less than 10.00
alpha-Linolenic acid (C18:3 ALA)	29.36
cis-11-Eicosenoic acid (C20:1)	43.10
Heneicosanoic acid (C21:0)	Less than 10.00
Stearidonic acid (18:4)	23.64
cis-11,14-Eicosadienoic acid (C20:2)	11.89
cis-5,8,11-Eicosatrienoic acid (C20:3 n-9)	Less than 10.00
Behenic acid (C22:0)	12.32
cis-8,11,14-Eicosatrienoic acid (C20:3 n-6)	Less than 10.00
trans-13-Docosenoic acid (C22:1t)	Less than 10.00
cis-11,14,17-Eicosatrienoic acid (C20:3 n-3)	11.01
cis-5,8,11,14-Eicosatetraenoic acid (C20:4 ARA)	77.89
Erucic acid (C22:1)	Less than 10.00
Tricosanoic acid (C23:0)	Less than 10.00
cis 8,11,14,17 Eicosatetraenoic acid C20:4(n-3)	16.64
cis-13,16-Docosadienoic acid (C22:2)	Less than 10.00
cis-5,8,11,14,17-Eicosapentaenoic acid (C20:5 EPA)	194.95
Lignoceric acid (C24:0)	11.25
cis-13,16,19-Docosatrienoic acid (C22:3)	Less than 10.00
Nervonic acid (C24:1)	19.18
Adrenic acid (C22:4)	12.50
Docosapentaenoic acid (C22:5)	75.28
cis-7,10,13,16,19-Docosapentaenoic acid (C22:5 DPA)	70.78
cis-4,7,10,13,16,19-Docosahexaenoic acid (C22:6 DHA)	948.37

Clinical study - End Intervention

SGS Test Report no.	Sample: 4 Formula 11 (Placebo) at end intervention, Sunflower oil-containing beverage in clinical study
5388895	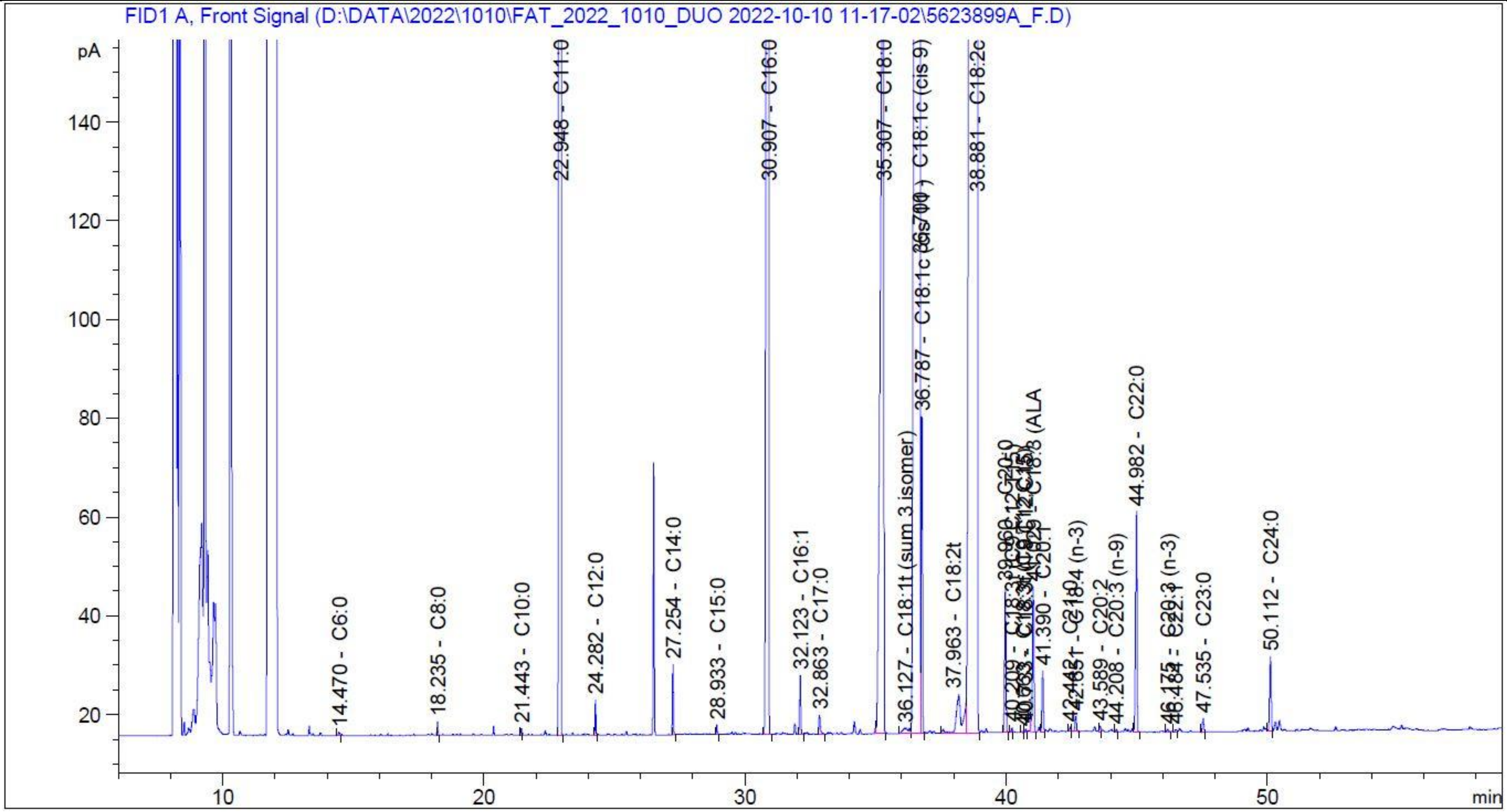

Total Fatty acid	mg/100ml
Total fatty acid	5885.12
Butyric acid (C4:0)	Less than 10.00
Caproic acid (C6:0)	Less than 10.00
Caprylic acid (C8:0)	Less than 10.00
Capric acid (C10:0)	Less than 10.00
Lauric acid (C12:0)	Less than 10.00
Tridecanoic acid (C13:0)	Less than 10.00
Myristic acid (C14:0)	Less than 10.00
Myristoleic acid (C14:1)	Less than 10.00

SGS Test Report no.	Sample: 4 Formula 11 (Placebo) at end intervention, Sunflower oil-containing beverage in clinical study

Pentadecanoic acid (C15:0)	Less than 10.00
cis-10-Pentadecenoic acid (C15:1)	Less than 10.00
Palmitic acid (C16:0)	564.37
trans-9-Hexadecenoic acid (C16:1t)	Less than 10.00
Palmitoleic acid (C16:1)	Less than 10.00
Heptadecanoic acid (C17:0)	Less than 10.00
cis-10-Heptadecenoic acid (C17:1)	Less than 10.00
Stearic acid (C18:0)	317.88
C18:1t (Sum3 isomer)	Less than 10.00
cis-9-Oleic acid (C18:1 c)	1609.28
cis-11-vacenic acid (C18:1c)	43.16
cis-12-octadecenoic acid (C18:1c)	Less than 10.00
trans-9,12-Octadecadienoic acid (C18:2t)	22.92
cis-9,12-Octadecadienoic acid (C18:2 c)	3232.98
18:3 trans-9, trans-12, trans-15-octadecatrienoic	Less than 10.00
Arachidic acid (C20:0)	21.39
18:3 trans-9, trans-12, cis-15-octadecatrienoic	Less than 10.00
18:3 trans-9, cis-12, trans-15-octadecatrienoic	Less than 10.00
Gamma-Linolenic acid (C18:3 GLA)	Less than 10.00
18:3 cis-9, trans-12, trans-15-octadecatrienoic	Less than 10.00
18:3 cis-9, cis-12, trans-15-octadecatrienoic	Less than 10.00
18:3 cis-9, trans-12, cis-15-octadecatrienoic	Less than 10.00
18:3 trans-9, cis-12, cis-15-octadecatrienoic	Less than 10.00
trans-11-Eicosenoic acid (C20:1t)	Less than 10.00
alpha-Linolenic acid (C18:3 ALA)	23.38
cis-11-Eicosenoic acid (C20:1)	Less than 10.00
Heneicosanoic acid (C21:0)	Less than 10.00
Stearidonic acid (18:4)	Less than 10.00
cis-11,14-Eicosadienoic acid (C20:2)	Less than 10.00
cis-5,8,11-Eicosatrienoic acid (C20:3 n-9)	Less than 10.00
Behenic acid (C22:0)	36.66
cis-8,11,14-Eicosatrienoic acid (C20:3 n-6)	Less than 10.00
trans-13-Docosenoic acid (C22:1t)	Less than 10.00
cis-11,14,17-Eicosatrienoic acid (C20:3 n-3)	Less than 10.00
cis-5,8,11,14-Eicosatetraenoic acid (C20:4 ARA)	Less than 10.00
Erucic acid (C22:1)	Less than 10.00
Tricosanoic acid (C23:0)	Less than 10.00
cis 8,11,14,17 Eicosatetraenoic acid C20:4(n-3)	Less than 10.00
cis-13,16-Docosadienoic acid (C22:2)	Less than 10.00
cis-5,8,11,14,17-Eicosapentaenoic acid (C20:5 EPA)	Less than 10.00
Lignoceric acid (C24:0)	13.10
cis-13,16,19-Docosatrienoic acid (C22:3)	Less than 10.00

SGS Test Report no.	Sample: 4 Formula 11 (Placebo) at end intervention, Sunflower oil-containing beverage in clinical study

Nervonic acid (C24:1)	Less than 10.00
Adrenic acid (C22:4)	Less than 10.00
Docosapentaenoic acid (C22:5)	Less than 10.00
cis-7,10,13,16,19-Docosapentaenoic acid (C22:5 DPA)	Less than 10.00
cis-4,7,10,13,16,19-Docosahexaenoic acid (C22:6 DHA)	Less than 10.00

Clinical study - End Intervention

SGS Test Report no.	Sample: 5 Formula 22 at end intervention, 2600 mg Tuna oil-containing beverage in clinical study
5388908	

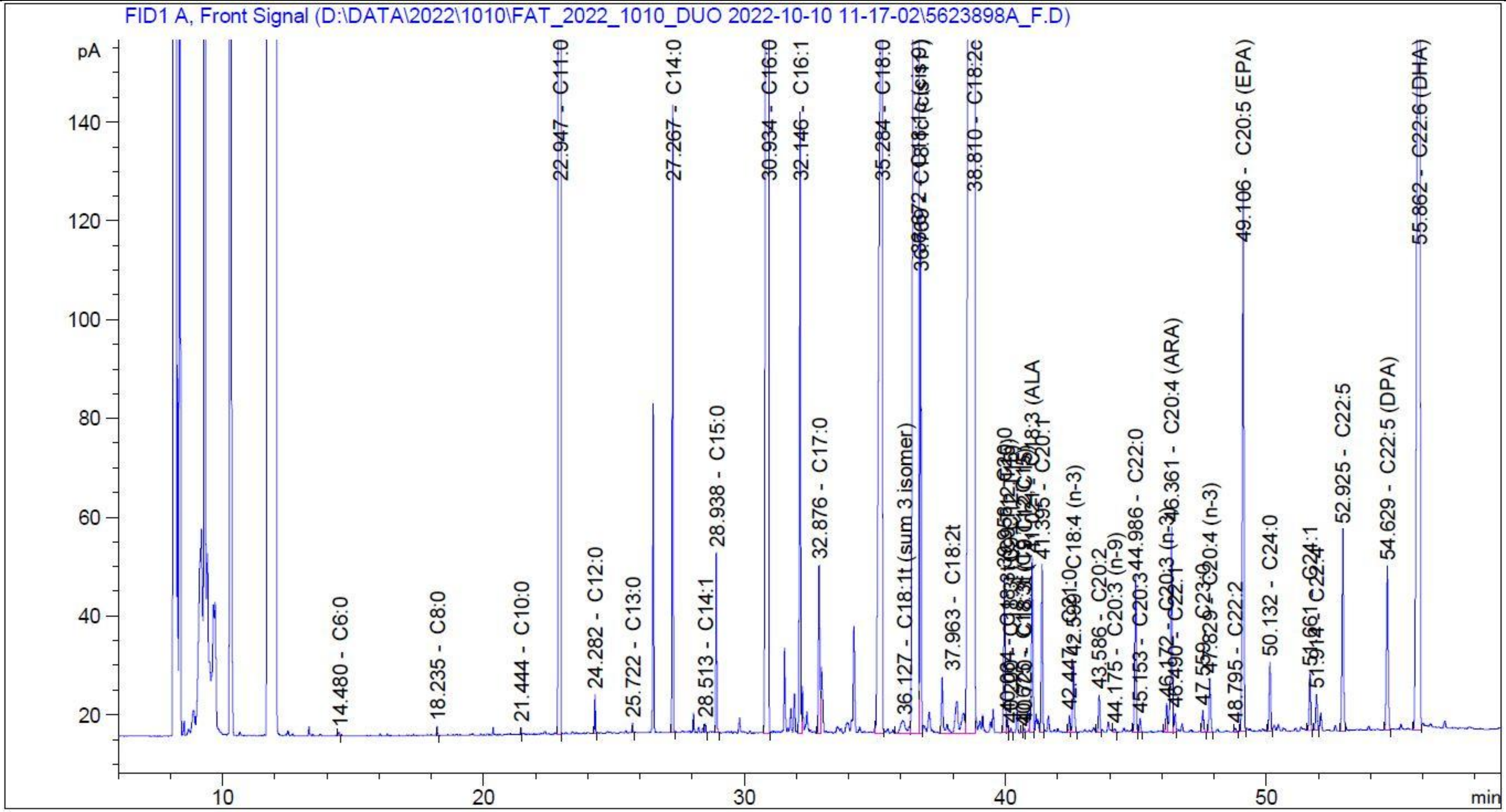

Total Fatty acid	mg/100ml
Total fatty acid	5658.22
Butyric acid (C4:0)	Less than 10.00
Caproic acid (C6:0)	Less than 10.00
Caprylic acid (C8:0)	Less than 10.00
Capric acid (C10:0)	Less than 10.00
Lauric acid (C12:0)	Less than 10.00
Tridecanoic acid (C13:0)	Less than 10.00
Myristic acid (C14:0)	61.92
Myristoleic acid (C14:1)	Less than 10.00

SGS Test Report no.	Sample: 5 Formula 22 at end intervention, 2600 mg Tuna oil-containing beverage in clinical study

Pentadecanoic acid (C15:0)	18.85
cis-10-Pentadecenoic acid (C15:1)	Less than 10.00
Palmitic acid (C16:0)	786.43
trans-9-Hexadecenoic acid (C16:1t)	Less than 10.00
Palmitoleic acid (C16:1)	78.19
Heptadecanoic acid (C17:0)	24.23
cis-10-Heptadecenoic acid (C17:1)	Less than 10.00
Stearic acid (C18:0)	352.51
C18:1t (Sum3 isomer)	Less than 10.00
cis-9-Oleic acid (C18:1 c)	1313.01
cis-11-vacenic acid (C18:1c)	67.53
cis-12-octadecenoic acid (C18:1c)	Less than 10.00
trans-9,12-Octadecadienoic acid (C18:2t)	28.16
cis-9,12-Octadecadienoic acid (C18:2 c)	2134.35
18:3 trans-9, trans-12, trans-15-octadecatrienoic	Less than 10.00
Arachidic acid (C20:0)	23.43
18:3 trans-9, trans-12, cis-15-octadecatrienoic	Less than 10.00
18:3 trans-9, cis-12, trans-15-octadecatrienoic	Less than 10.00
Gamma-Linolenic acid (C18:3 GLA)	Less than 10.00
18:3 cis-9, trans-12, trans-15-octadecatrienoic	Less than 10.00
18:3 cis-9, cis-12, trans-15-octadecatrienoic	Less than 10.00
18:3 cis-9, trans-12, cis-15-octadecatrienoic	Less than 10.00
18:3 trans-9, cis-12, cis-15-octadecatrienoic	Less than 10.00
trans-11-Eicosenoic acid (C20:1t)	Less than 10.00
alpha-Linolenic acid (C18:3 ALA)	27.77
cis-11-Eicosenoic acid (C20:1)	25.64
Heneicosanoic acid (C21:0)	Less than 10.00
Stearidonic acid (18:4)	12.23
cis-11,14-Eicosadienoic acid (C20:2)	Less than 10.00
cis-5,8,11-Eicosatrienoic acid (C20:3 n-9)	Less than 10.00
Behenic acid (C22:0)	26.63
cis-8,11,14-Eicosatrienoic acid (C20:3 n-6)	Less than 10.00
trans-13-Docosenoic acid (C22:1t)	Less than 10.00
cis-11,14,17-Eicosatrienoic acid (C20:3 n-3)	Less than 10.00
cis-5,8,11,14-Eicosatetraenoic acid (C20:4 ARA)	37.66
Erucic acid (C22:1)	Less than 10.00
Tricosanoic acid (C23:0)	Less than 10.00
cis 8,11,14,17 Eicosatetraenoic acid C20:4(n-3)	Less than 10.00
cis-13,16-Docosadienoic acid (C22:2)	Less than 10.00
cis-5,8,11,14,17-Eicosapentaenoic acid (C20:5 EPA)	94.38
Lignoceric acid (C24:0)	12.13
cis-13,16,19-Docosatrienoic acid (C22:3)	Less than 10.00

SGS Test Report no.	Sample: 5 Formula 22 at end intervention, 2600 mg Tuna oil-containing beverage in clinical study

Nervonic acid (C24:1)	10.11
Adrenic acid (C22:4)	Less than 10.00
Docosapentaenoic acid (C22:5)	35.18
cis-7,10,13,16,19-Docosapentaenoic acid (C22:5 DPA)	30.07
cis-4,7,10,13,16,19-Docosahexaenoic acid (C22:6 DHA)	457.81

Clinical study - End Intervention

SGS Test Report no.	Sample: 6 Formula 33 at end intervention, 6000 mg Tuna oil-containing beverage in clinical study
5388907	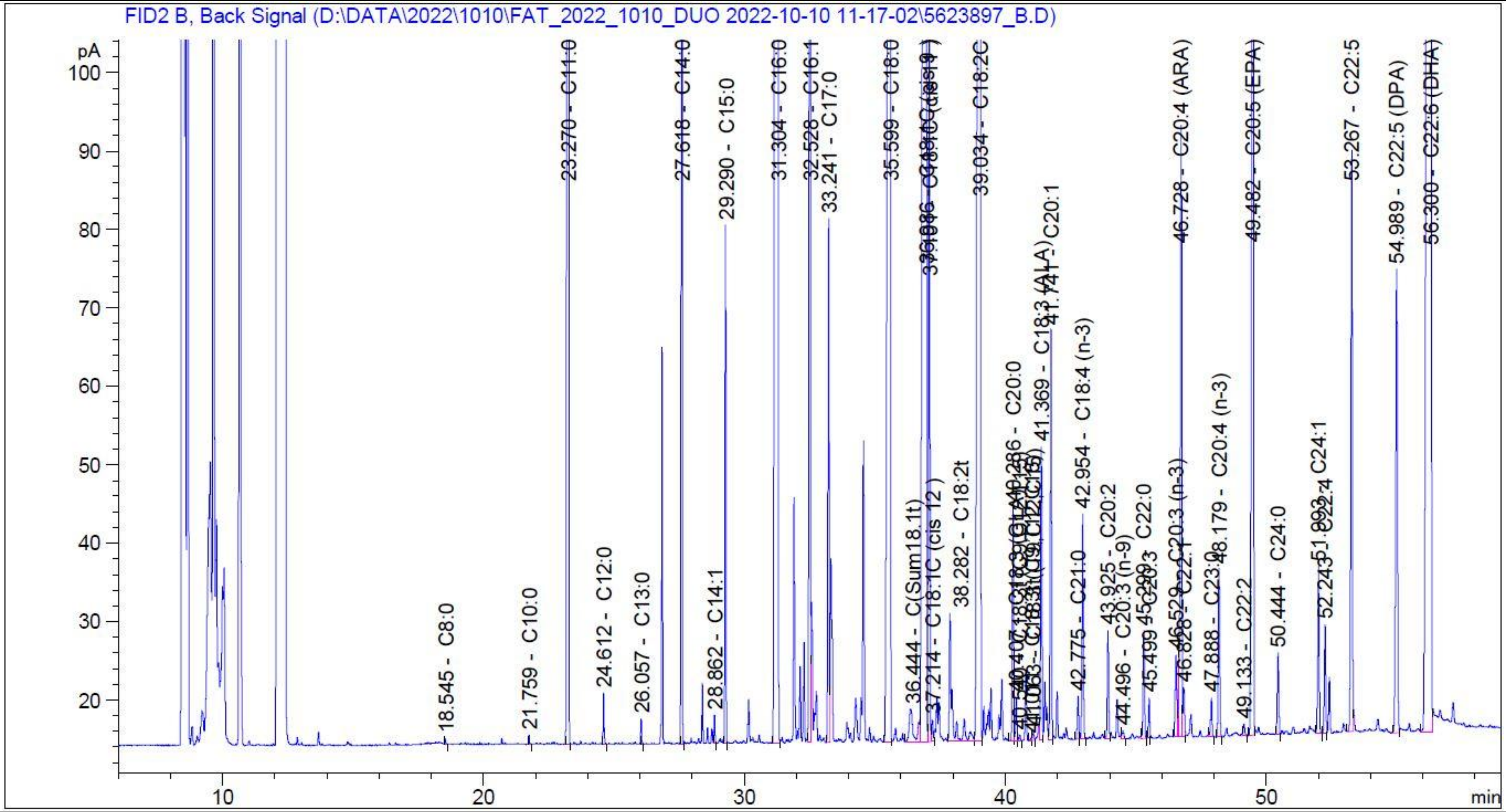

Total Fatty acid	mg/100ml
Total fatty acid	4942.06
Butyric acid (C4:0)	Less than 10.00
Caproic acid (C6:0)	Less than 10.00
Caprylic acid (C8:0)	Less than 10.00
Capric acid (C10:0)	Less than 10.00
Lauric acid (C12:0)	Less than 10.00
Tridecanoic acid (C13:0)	Less than 10.00
Myristic acid (C14:0)	121.16
Myristoleic acid (C14:1)	Less than 10.00

SGS Test Report no.	Sample: 6 Formula 33 at end intervention, 6000 mg Tuna oil-containing beverage in clinical study

Pentadecanoic acid (C15:0)	38.01
cis-10-Pentadecenoic acid (C15:1)	Less than 10.00
Palmitic acid (C16:0)	973.82
trans-9-Hexadecenoic acid (C16:1t)	Less than 10.00
Palmitoleic acid (C16:1)	155.32
Heptadecanoic acid (C17:0)	49.08
cis-10-Heptadecenoic acid (C17:1)	Less than 10.00
Stearic acid (C18:0)	362.50
C18:1t (Sum3 isomer)	Less than 10.00
cis-9-Oleic acid (C18:1 c)	889.67
cis-11-vacenic acid (C18:1c)	90.63
cis-12-octadecenoic acid (C18:1c)	Less than 10.00
trans-9,12-Octadecadienoic acid (C18:2t)	27.86
cis-9,12-Octadecadienoic acid (C18:2 c)	671.30
18:3 trans-9, trans-12, trans-15-octadecatrienoic	Less than 10.00
Arachidic acid (C20:0)	23.74
18:3 trans-9, trans-12, cis-15-octadecatrienoic	Less than 10.00
18:3 trans-9, cis-12, trans-15-octadecatrienoic	Less than 10.00
Gamma-Linolenic acid (C18:3 GLA)	Less than 10.00
18:3 cis-9, trans-12, trans-15-octadecatrienoic	Less than 10.00
18:3 cis-9, cis-12, trans-15-octadecatrienoic	Less than 10.00
18:3 cis-9, trans-12, cis-15-octadecatrienoic	Less than 10.00
18:3 trans-9, cis-12, cis-15-octadecatrienoic	Less than 10.00
trans-11-Eicosenoic acid (C20:1t)	Less than 10.00
alpha-Linolenic acid (C18:3 ALA)	31.23
cis-11-Eicosenoic acid (C20:1)	43.23
Heneicosanoic acid (C21:0)	Less than 10.00
Stearidonic acid (18:4)	22.83
cis-11,14-Eicosadienoic acid (C20:2)	11.77
cis-5,8,11-Eicosatrienoic acid (C20:3 n-9)	Less than 10.00
Behenic acid (C22:0)	12.58
cis-8,11,14-Eicosatrienoic acid (C20:3 n-6)	Less than 10.00
trans-13-Docosenoic acid (C22:1t)	Less than 10.00
cis-11,14,17-Eicosatrienoic acid (C20:3 n-3)	11.56
cis-5,8,11,14-Eicosatetraenoic acid (C20:4 ARA)	72.67
Erucic acid (C22:1)	Less than 10.00
Tricosanoic acid (C23:0)	Less than 10.00
cis 8,11,14,17 Eicosatetraenoic acid C20:4(n-3)	15.79
cis-13,16-Docosadienoic acid (C22:2)	Less than 10.00
cis-5,8,11,14,17-Eicosapentaenoic acid (C20:5 EPA)	197.80
Lignoceric acid (C24:0)	Less than 10.00
cis-13,16,19-Docosatrienoic acid (C22:3)	Less than 10.00

SGS Test Report no.	Sample: 6 Formula 33 at end intervention, 6000 mg Tuna oil-containing beverage in clinical study

Nervonic acid (C24:1)	19.82
Adrenic acid (C22:4)	12.78
Docosapentaenoic acid (C22:5)	73.05
cis-7,10,13,16,19-Docosapentaenoic acid (C22:5 DPA)	63.26
cis-4,7,10,13,16,19-Docosahexaenoic acid (C22:6 DHA)	950.60
