# Supplementary material for: A Randomized, Double-Blind, Placebo-Controlled, Parallel-Group, 8-Week Pilot Study of Tuna-Byproduct-Derived Novel Supplements for Managing Cellular Senescence and Cognitive Decline in Perimenopausal and Postmenopausal Women
Source: Antioxidants (Basel). 2025 Apr 27;14(5):520. doi: 10.3390/antiox14050520 (PMC12108292; doi:10.3390/antiox14050520)
Supplement: Supplementary file 1 [file antioxidants-14-00520-s001.zip › S3 FFQ .pdf]

Table S3-1 Food frequency questionnaire of adulthood volunteers who consumed placebo, or the functional drink at the doses of 2600, and 6000 mg per day at baseline. (N=20/arm) Data are expressed as mean±S.E.M.

Parameters	Treatment group	Baseline
**Rice cooked (scoop)**	Placebo	30.10 ± 3.63
	Functional drink at the dose of 2,600 mg/day	27.23 ± 3.87 (p=0.284)
	Functional drink at the dose of 6,000 mg/day	23.55 ± 1.68 (p=0.066)
**Meat (piece)**	Placebo	61.81 ± 8.83
	Functional drink at the dose of 2,600 mg/day	70.38 ± 11.07 (p=0.553)
	Functional drink at the dose of 6,000 mg/day	51.33 ± 9.73 (p=0.477)
**Egg**	Placebo	14.90 ± 4.62
	Functional drink at the dose of 2,600 mg/day	9.38 ± 1.60 (p=0.384)
	Functional drink at the dose of 6,000 mg/day	7.75 ± 1.39 (p=0.147)
**Milk (glass/box)**	Placebo	8.45 ± 2.03
	Functional drink at the dose of 2,600 mg/day	3.07 ± 0.91 (p=0.075)
	Functional drink at the dose of 6,000 mg/day	8.47 ±1.41(p=0.226)
**Vegetables (cup)**	Placebo	16.04 ± 2.73
	Functional drink at the dose of 2,600 mg/day	13.84 ± 1.39 (p=0.642)
	Functional drink at the dose of 6,000 mg/day	13.62 ± 1.78 (p=0.663)
**Fruit (cup)**	Placebo	11.72 ± 3.51
	Functional drink at the dose of 2,600 mg/day	9.38 ± 1.81 (p=0.771)
	Functional drink at the dose of 6,000 mg/day	9.95 ± 1.44 (p=1.000)
**Tuna meat (cup)**	Placebo	1.50 ± 0.61
	Functional drink at the dose of 2,600 mg/day	0.15 ± 0.10 (p=0.065)
	Functional drink at the dose of 6,000 mg/day	0.58 ± 0.43 (p=0.151)
**Salmon meat (cup)**	Placebo	0.31 ± 0.31
	Functional drink at the dose of 2,600 mg/day	0.00 ± 0.00 (p=0.277)
	Functional drink at the dose of 6,000 mg/day	0.08 ± 0.08 (p=0.900)
**Fish oil/tablet/day**	Placebo	0.00 ± 0.00
	Functional drink at the dose of 2,600 mg/day	0.00 ± 0.00 (p=1.000)
	Functional drink at the dose of 6,000 mg/day	0.36 ± 0.20 (p=0.069)
**Canned tuna/cans/day**	Placebo	0.45 ± 0.45
	Functional drink at the dose of 2,600 mg/day	0.07 ± 0.07 (p=0.856)
	Functional drink at the dose of 6,000 mg/day	0.16 ± 0.16 (p=0.900)

Table S-2 Food frequency questionnaire of adulthood volunteers who consumed placebo, or the functional drink at the doses of 2600, and 6000 mg per day after 4-week of consumption. (N=20/arm) Data are expressed as mean±S.E.M.

Parameters	Treatment group	4-week
Rice cooked (scoop)	Placebo	33.72 ± 5.09
	Functional drink at the dose of 2,600 mg/day	31.23 ± 3.89 (p=0.682)
	Functional drink at the dose of 6,000 mg/day	30.45 ± 3.94 (p=0.458)
Meat (piece)	Placebo	67.27 ± 14.21
	Functional drink at the dose of 2,600 mg/day	79.46 ± 14.82 (p=0.562)
	Functional drink at the dose of 6,000 mg/day	60.08 ± 12.33 (p=0.805)
Egg	Placebo	11.36 ± 1.85
	Functional drink at the dose of 2,600 mg/day	9.92 ± 1.51 (p=0.550)
	Functional drink at the dose of 6,000 mg/day	10.83 ± 1.70 (p=0.829)
Milk (glass/box)	Placebo	4.63 ± 1.53
	Functional drink at the dose of 2,600 mg/day	5.23 ± 1.60 (p=0.859)
	Functional drink at the dose of 6,000 mg/day	7.00 ± 1.96 (p=0.348)
Vegetables (cup)	Placebo	11.04 ± 2.32
	Functional drink at the dose of 2,600 mg/day	12.73 ± 2.03 (p=0.641)
	Functional drink at the dose of 6,000 mg/day	13.12 ± 1.78 (p=0.403)
Fruit (cup)	Placebo	7.72 ± 1.80
	Functional drink at the dose of 2,600 mg/day	10.23 ± 1.70 (p=0.357)
	Functional drink at the dose of 6,000 mg/day	13.08 ± 2.13 (p=0.058)
Tuna meat (cup)	Placebo	2.00 ± 1.20
	Functional drink at the dose of 2,600 mg/day	0.15 ± 0.10 (p=0.170)
	Functional drink at the dose of 6,000 mg/day	0.41 ± 0.28 (p=0.248)
Salmon meat (cup)	Placebo	0.09 ± 0.09
	Functional drink at the dose of 2,600 mg/day	0.07 ± 0.07 (p=0.904)
	Functional drink at the dose of 6,000 mg/day	0.00 ± 0.00 (p=0.296)
Fish oil/tablet/day	Placebo	0.00 ± 0.00
	Functional drink at the dose of 2,600 mg/day	0.00 ± 0.00 (p=1.000)
	Functional drink at the dose of 6,000 mg/day	1.16 ± 0.78 (p=0.166)
Canned tuna/cans/day	Placebo	1.90 ± 1.32
	Functional drink at the dose of 2,600 mg/day	0.07 ± 0.07 (p=0.392)
	Functional drink at the dose of 6,000 mg/day	0.16 ± 0.16 (p=0.431)

Table S3-3 Food frequency questionnaire of adulthood volunteers who consumed placebo, or the functional drink at the doses of 2600, and 6000 mg per day after 8 weeks of consumption. (N=20/arm) Data are expressed as mean±S.E.M.

**Parameters**	**Treatment group**	**8-week**
Rice cooked (scoop)	Placebo	33.45 ± 5.62
	Functional drink at the dose of 2,600 mg/day	31.30 ± 3.83 (p=0.907)
	Functional drink at the dose of 6,000 mg/day	27.50 ± 4.83 (p=0.537)
Meat (piece)	Placebo	56.09 ± 11.03
	Functional drink at the dose of 2,600 mg/day	63.76 ± 16.51 (p=0.862)
	Functional drink at the dose of 6,000 mg/day	40.91 ± 11.12 (p=0.183)
Egg	Placebo	9.81 ± 2.21
	Functional drink at the dose of 2,600 mg/day	12.84 ± 3.20 (p=0.954)
	Functional drink at the dose of 6,000 mg/day	12.16 ± 2.18 (p=0.497)
Milk (glass/box)	Placebo	5.31 ± 1.60
	Functional drink at the dose of 2,600 mg/day	5.30 ± 2.04 (p=0.692)
	Functional drink at the dose of 6,000 mg/day	6.00 ± 1.80 (p=0.851)
Vegetables (cup)	Placebo	10.50 ± 1.46
	Functional drink at the dose of 2,600 mg/day	11.92 ± 2.01 (p=0.503)
	Functional drink at the dose of 6,000 mg/day	15.91 ± 2.54 (p=0.076)
Fruit (cup)	Placebo	9.45 ± 1.61
	Functional drink at the dose of 2,600 mg/day	10.38 ± 2.28 (p=0.745)
	Functional drink at the dose of 6,000 mg/day	12.66 ± 1.90 (p=0.274)
Tuna meat (cup)	Placebo	1.18 ± 0.77
	Functional drink at the dose of 2,600 mg/day	0.30 ± 0.17 (p=0.675)
	Functional drink at the dose of 6,000 mg/day	1.25 ± 0.60 (p=0.587)
Salmon meat (cup)	Placebo	0.45 ± 0.36
	Functional drink at the dose of 2,600 mg/day	0.00 ± 0.00 (p=0.116)
	Functional drink at the dose of 6,000 mg/day	0.33 ± 0.33 (p=0.528)
Fish oil/tablet/day	Placebo	0.00 ± 0.00
	Functional drink at the dose of 2,600 mg/day	0.53 ± 0.53 (p=0.358)
	Functional drink at the dose of 6,000 mg/day	0.75 ± 0.75 (p=0.338)
Canned tuna/cans/day	Placebo	0.45 ± 0.24
	Functional drink at the dose of 2,600 mg/day	0.07 ± 0.07 (p=0.180)
	Functional drink at the dose of 6,000 mg/day	0.41 ± 0.22 (p=0.904)
