# Supplementary material for: A Randomized, Double-Blind, Placebo-Controlled, Parallel-Group, 8-Week Pilot Study of Tuna-Byproduct-Derived Novel Supplements for Managing Cellular Senescence and Cognitive Decline in Perimenopausal and Postmenopausal Women
Source: Antioxidants (Basel). 2025 Apr 27;14(5):520. doi: 10.3390/antiox14050520 (PMC12108292; doi:10.3390/antiox14050520)
Supplement: Supplementary file 1 [file antioxidants-14-00520-s001.zip › S4 Nutritional profile of tuna oil containing beverages and the placebo.pdf]

Nutritional profile of tuna oil-containing beverages and the placebo

| Nutrient per 150 ml | Placebo (Code 11) | Tuna oil-containing beverages (Tuna oil 2,600 mg) (Code 12) | Tuna oil-containing beverages (Tuna oil 6,000 mg) (Code 13) |
|---|---|---|---|
| Energy (Kcal) | 130 | 130 | 130 |
| Protein (g) | 7 | 7 | 7 |
| Carbohydrate (g) | 10 | 10 | 10 |
| Sugar (g) | 8 | 8 | 8 |
| Fat (g) | 7 | 7 | 7 |
| DHA (mg) | ND | 600 | 1400 |
| EPA (mg) | ND | 120 | 300 |
| Sodium (mg) | 120 | 120 | 120 |
| Vitamin B1 (mg) | 1.05 | 1.05 | 1.05 |
| Vitamin B 2 (mg) | 0.85 | 0.85 | 0.85 |
| Niacin (mg NE) | 4 | 4 | 4 |
| Pantothenic Acid (mg) | 3 | 3 | 3 |
| Vitamin B 6 (mg) | 1.2 | 1.2 | 1.2 |
| Biotin (ug) | 60 | 60 | 60 |
| Folate (ug) | 80 | 80 | 80 |
| Vitamin B 12 (ug) | 1.6 | 1.6 | 1.6 |
| Vitamin E (mg alpha-TE) | 8 | 8 | 8 |
