# Supplementary material for: A Randomized, Double-Blind, Placebo-Controlled, Parallel-Group, 8-Week Pilot Study of Tuna-Byproduct-Derived Novel Supplements for Managing Cellular Senescence and Cognitive Decline in Perimenopausal and Postmenopausal Women
Source: Antioxidants (Basel). 2025 Apr 27;14(5):520. doi: 10.3390/antiox14050520 (PMC12108292; doi:10.3390/antiox14050520)
Supplement: Supplementary file 1 [file antioxidants-14-00520-s001.zip › S5 Nucleic acid sequence.pdf]

Supplementary material S3 :Nucleic sequence of Telomerase Reverse Transcriptase (TERT), and Telomerase RNA Component (TERC)

Telomerase Reverse Transcriptase (TERT): The protein component responsible for adding DNA sequence repeats to the telomere ends.

Gene Symbol: TERT

Gene ID: 7015

RefSeq Accession Number: NM_198253.3

UniProt Accession Number: O14746

GenBank Accession Number for Genomic Sequence: AY007685

NATURE.COM

Telomerase RNA Component (TERC): The RNA template that TERT uses to synthesize telomeric DNA repeats.

Gene Symbol: TERC

Gene ID: 7012

RefSeq Accession Number: NR_001566.1

GenBank Accession Number: U86046

ACADEMIC.OUP.COM
